# Carbapenem Resistance in India: A Systematic Review of Epidemiology, Diagnostic Strategies, and Therapeutic Approaches

**DOI:** 10.1155/bmri/6806493

**Published:** 2026-08-12

**Authors:** Akhil Kapoor, Anuj Gupta, Bipinesh Sansar, Bal Krishna Mishra, Madhu Singh, Rahul Sarode, Vijeta Bajpai, Anil Singh, Sujit Bharti, Anwita Mishra, Ankita Chaurasia, Satyajit Pradhan

**Affiliations:** ^1^ Departments of Medical Oncology, Mahamana Pandit Madan Mohan Malaviya Cancer Centre and Homi Bhabha Cancer Hospital, Tata Memorial Centre, Homi Bhabha National Institute, Varanasi, India, hbni.ac.in; ^2^ Departments of Medical Microbiology, Mahamana Pandit Madan Mohan Malaviya Cancer Centre and Homi Bhabha Cancer Hospital, Tata Memorial Centre, Homi Bhabha National Institute, Varanasi, India, hbni.ac.in; ^3^ Departments of Medical Radiation Oncology, Mahamana Pandit Madan Mohan Malaviya Cancer Centre and Homi Bhabha Cancer Hospital, Tata Memorial Centre, Homi Bhabha National Institute, Varanasi, India, hbni.ac.in

**Keywords:** antimicrobial stewardship, carbapenem resistance, India, NDM, OXA-48, PRISMA 2020, systematic review

## Abstract

**Background:**

Carbapenem‐resistant gram‐negative infections pose a major public health challenge in India due to high antibiotic consumption, limited diagnostic access, and regional variation in resistance mechanisms. We systematically reviewed recent data to evaluate prevalence, diagnostics, and therapeutic strategies.

**Methods:**

Following PRISMA 2020 guidelines, we searched PubMed, Scopus, Embase, and Google Scholar for studies (January 2019–December 2024) reporting carbapenem resistance in clinical gram‐negative isolates from India. Data on prevalence, molecular mechanisms, diagnostics, and treatment outcomes were extracted. Study quality was assessed using the Joanna Briggs Institute (JBI) Checklist.

**Results:**

From 75 studies (> 25,000 isolates), pooled resistance rates were *K. pneumoniae* 42.3%, *E. coli* 35.6%, *A. baumannii* 58.9%, and *P. aeruginosa* 41.7%. NDM (65%) and OXA‐48‐like (20%) enzymes predominated. Only 38% of studies employed rapid molecular diagnostics. Treatment strategies included ceftazidime‐avibactam ± aztreonam, colistin‐based regimens, and cefiderocol, but outcomes were inconsistent.

**Conclusions:**

Carbapenem resistance in India remains alarmingly high, primarily driven by NDM and OXA‐type carbapenemases. Optimised diagnostics, improved access to effective antimicrobials, robust antimicrobial stewardship, and national surveillance are critical to mitigate its growing clinical and public health impact.

## 1. Introduction

Carbapenem‐resistant gram‐negative bacteria (CR‐GNB) represent one of the most pressing threats to global public health, driven by escalating antimicrobial resistance (AMR) and limited therapeutic options. The World Health Organization has classified carbapenem‐resistant Enterobacterales (CRE), *Acinetobacter baumannii*, and *Pseudomonas aeruginosa* as critical priority pathogens, underscoring the urgent need for new diagnostics and therapeutics [1]. The global burden of AMR‐associated mortality is substantial, with an estimated 4.95 million deaths linked to bacterial AMR in 2019, of which carbapenem‐resistant organisms contributed significantly [2].

Carbapenems have historically served as last‐resort agents for multidrug‐resistant gram‐negative infections. However, their efficacy has been severely compromised due to the emergence of diverse resistance mechanisms, particularly carbapenemase enzymes such as New Delhi metallo‐*β*‐lactamase (NDM), OXA‐type enzymes, and *Klebsiella pneumoniae* carbapenemase (KPC) [3, 4]. In addition to enzymatic degradation, nonenzymatic mechanisms—including porin loss, efflux pump overexpression, and target site modifications—play a crucial role in mediating resistance and often act synergistically with carbapenemases to confer high‐level resistance [5, 6].

India represents a major epicenter of carbapenem resistance due to a combination of factors, including high antibiotic consumption, inadequate antimicrobial stewardship, limited access to rapid diagnostics, and challenges in infection prevention practices [6]. Since the first description of NDM‐1 in 2008, there has been rapid dissemination of carbapenem‐resistant organisms across healthcare and community settings [6]. Recent surveillance data indicate that resistance rates among *K. pneumoniae* and *A. baumannii* frequently exceed 50% in tertiary care centers [7–10].

The clinical implications are profound, with carbapenem‐resistant infections associated with increased mortality, prolonged hospitalization, and higher healthcare costs [4]. Therapeutic options remain limited and often rely on toxic or less effective agents such as polymyxins, though newer *β*‐lactam/*β*‐lactamase inhibitor combinations and agents like cefiderocol have shown promise [11, 12].

Given the rapidly evolving epidemiology and therapeutic landscape, there is a critical need to synthesize contemporary evidence on carbapenem resistance in India. Therefore, this systematic review aims to evaluate the epidemiology comprehensively, underlying resistance mechanisms (both enzymatic and nonenzymatic), diagnostic approaches, and therapeutic strategies for carbapenem‐resistant gram‐negative infections in India.

## 2. Methods

The review strictly adhered to PRISMA 2020 guidelines, including structured search strategy, predefined inclusion criteria, dual‐reviewer screening, and systematic data extraction, ensuring methodological rigor consistent with systematic review standards. A comprehensive literature search was performed (in PubMed, Scopus, and Google Scholar) to identify studies published from January 2019 through December 2024 that reported on carbapenem resistance in India. The search strategy was expanded to explicitly include organism‐specific keywords such as “*Acinetobacter baumannii*” and “*Pseudomonas aeruginosa*” to ensure comprehensive capture of studies involving nonfermenting gram‐negative pathogens. We included observational studies (cohort, case‐control, cross‐sectional, case series) and surveillance reports that provided data on prevalence, molecular mechanisms, clinical outcomes, or management of carbapenem‐resistant infections in Indian settings. Only English‐language articles were included. Two reviewers independently screened titles/abstracts and full texts for eligibility. Data on study setting, design, sample size, bacterial species, carbapenem resistance rates, resistance mechanisms (e.g., carbapenemase genes), and key findings were extracted using a standardized form.

Study quality was assessed using the Joanna Briggs Institute (JBI) Critical Appraisal Checklists for observational studies, tailored to study design (cross‐sectional, cohort, and case‐control studies), enabling systematic evaluation of methodological rigor and risk of bias.

Data extraction was performed using a predefined standardized data extraction form developed in Microsoft Excel, capturing variables including study design, geographic region, sample size, pathogen type, resistance rates, molecular mechanisms, diagnostic methods, and treatment outcomes. The form was pilot‐tested on a subset of studies to ensure consistency.

**Figure 1:**
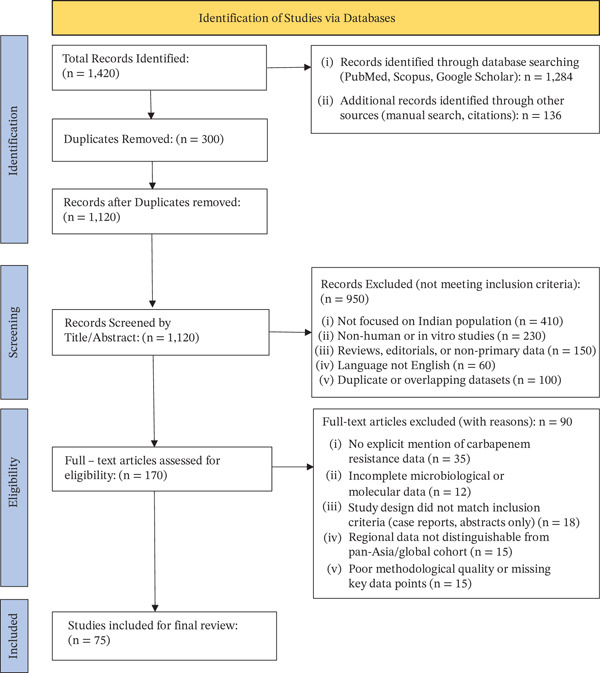
PRISMA 2020 flow diagram–study selection process.

The study selection process is summarized in the PRISMA flow diagram (Figure 1). Of the 1420 records initially identified, 75 studies met the inclusion criteria after removing duplicates and exclusion of ineligible articles. We compiled a PRISMA checklist (Table S1) to ensure transparency of methods. Any discrepancies in study inclusion or data extraction were resolved by consensus among the authors.

## 3. Results

### 3.1. Epidemiology of Carbapenem Resistance in India (2019–2024)

#### 3.1.1. Included Studies

We ultimately included 75 studies spanning various regions of India between 2019 and 2024. These studies encompassed tertiary‐care hospital surveillance reports, multicentre cohort studies, and a few nationwide data analyses. Most studies focused on healthcare‐associated infections due to carbapenemase‐producing Enterobacterales (CRE) and other CR‐GNB.

#### 3.1.2. Rising Prevalence

A consistent finding was the alarming increase in carbapenem resistance rates in India over the past 5 years. For instance, multiple hospital‐based studies reported that the proportion of Enterobacterales (particularly *K. pneumoniae* and *Escherichia coli*) nonsusceptible to carbapenems rose from roughly 10% in the late 2000s to around 50%–60% by 2023–2024 in their settings [7, 13–15]. One longitudinal analysis from a tertiary care center in eastern India found that overall carbapenem resistance in Enterobacterales had reached ~60% by 2024, up from 9% in 2008 [13]. Similar trends were observed among nonfermenting bacteria, with carbapenem resistance rates in *A. baumannii* exceeding 50% in several studies based on intensive care unit (ICU) [16,17], whereas resistance in *P. aeruginos*a increased to 20%–30% or higher across multiple reports [18]. Most carbapenem‐resistant infections were nosocomial (hospital‐acquired), with especially high incidence in ICUs and other critical‐care areas [19–21]. Notably, a study of ICU bloodstream infections at a South Indian center documented extremely high mortality (up to 68%) among patients with carbapenem‐resistant *K. pneumoniae* sepsis [4]. Such grim outcomes underscore the urgent public health challenge posed by these pathogens in India.

#### 3.1.3. Geographical Variation

The burden of carbapenem‐resistant organisms varies by region within India, though it is uniformly high. Surveillance data indicate that urban tertiary hospitals tend to report higher CRE prevalence compared to smaller or rural healthcare centers [8, 22, 23]. This likely reflects both greater antibiotic selective pressure in large hospitals and better laboratory detection capabilities. Nonetheless, carbapenem resistance is rising even in community and rural settings, eroding what was previously a predominantly hospital‐centric problem [24]. Among the included studies, North and South Indian tertiary centers reported some of the highest CRE rates [20, 25, 26], but worrying increases were also noted from East and Central India by 2023 [27–29]. The COVID‐19 pandemic (2020–2021) may have further exacerbated resistance trends, given increased antibiotic use and prolonged hospital stays, although robust data on COVID‐related impacts were limited in the review [30].

#### 3.1.4. Major Pathogens

Across studies, carbapenem‐resistant *K. pneumoniae* emerged as the most frequently reported pathogen, followed by carbapenem‐resistant *A. baumannii* [9, 10, 16, 17]. Together, these two accounted for a majority of carbapenem‐resistant gram‐negative infections in many hospital series. *K. pneumoniae* is a leading cause of CRE bacteraemia, pneumonia, and urinary tract infections in India [7], whereas *A. baumannii* predominates in ventilator‐associated pneumonias and wound infections in ICU [16, 31]. Carbapenem‐resistant *E. coli* (often carrying similar resistance genes as *Klebsiella*) was also commonly reported, especially in urinary and intra‐abdominal infections [32, 33], though at slightly lower rates than *K. pneumoniae* in most settings. *P. aeruginosa* showed high resistance to carbapenems as well [18], though some studies noted its rates increased more gradually compared to *K. pneumoniae* and *A. baumanni*. Importantly, coinfection or co‐colonization with multiple carbapenem‐resistant organisms (e.g., *K. pneumoniae* plus *A. baumanni* in the same patient) was occasionally described in outbreak settings, complicating treatment and infection control [34].

### 3.2. Molecular Mechanisms of Resistance

#### 3.2.1. Carbapenemase Enzymes

The predominant mechanism driving carbapenem resistance in Indian isolates is the production of carbapenemase enzymes that hydrolyse the carbapenem group of antibiotics. A wide array of carbapenemases has been identified in India, but metallo‐*β*‐lactamases (MBLs) of the NDM family are by far the most prevalent. Numerous studies confirm that bla_NDM-1_ (and its variants, such as blaNDM‐5) is the single most common carbapenemase found in CRE from India [8, 28, 35]. For example, Indrajith et al. (2020) reported NDM‐type enzymes in a majority of carbapenem‐resistant *K. pneumoniae* from clinical samples across India [13]. OXA‐type carbapenemases are the next most frequent: *OXA-48-like enzymes* (such as bla_OXA-48_, bla_OXA-181_, and bla_OXA-232_) have been widely detected in Indian CRE [25, 36–38]. A recent study by Das et al. characterized emerging _blaOXA-232_‐producing *K. pneumoniae* strains, highlighting the spread of this bla_OXA-48_ variant in India [8]. In contrast, KPC‐type enzymes – which dominate carbapenem resistance in the West – remain relatively uncommon in India, though sporadic cases exist [28]. A few reports also documented VIM and IMP Metallo‐enzymes in *P. aeruginosa* or *A. baumanni* [18, 38], but these are less common than bla_NDM_ in the Indian context.

#### 3.2.2. Co‐production and Plasmids

Alarmingly, Indian CRE isolates often harbor multiple resistance genes simultaneously. Co‐production of an NDM Metallo‐*β*‐lactamase together with an OXA‐48‐like enzyme in the same strain has been increasingly reported in recent years [34, 39]. This dual‐carbapenemase scenario is especially problematic, as it can confer high‐level resistance to virtually all *β*‐lactams and even compromise some of the newest *β*‐lactamase inhibitor combinations. Many carbapenemase genes are carried on plasmids and other mobile genetic elements, facilitating their spread between bacteria. Plasmid‐mediated dissemination of bla_NDM-1_ from India has been well‐documented since its discovery [26, 35] and continues to drive international spread of CRE. Besides carbapenemases, plasmids often carry additional *β*‐lactamase genes (e.g., for extended‐spectrum *β*‐lactamases [ESBLs]), further broadening resistance [32, 40]. The propensity of *K. pneumoniae* (in particular) to acquire multiple resistance determinants—a phenomenon noted in genomics studies of Indian isolates [10, 11]—creates multidrug‐resistant clones adept at surviving heavy antibiotic pressure.

#### 3.2.3. Porin Loss and Efflux

In some carbapenem‐resistant strains, nonenzymatic mechanisms augment the resistance conferred by carbapenemases. Common examples include porin mutations and efflux pump overexpression. *K. pneumoniae* frequently has mutations or deletions in its outer membrane porins (OmpK35, OmpK36), which reduce entry of carbapenems into the bacterial cell [10]. This porin loss, when combined with even low‐level carbapenemase production, yields significantly higher resistance levels [5]. Similarly, upregulation of efflux pumps that expel antibiotics can contribute to carbapenem resistance. Although difficult to quantify clinically, research has shown overexpression of efflux genes (like *acrAB* in Enterobacterales) in resistant isolates from India [5, 41], suggesting a role in multidrug resistance phenotypes. These additional mechanisms do not usually cause carbapenem resistance on their own, but they compound the effect of carbapenemases, making the bacteria more refractory to treatment. Adaptive regulatory changes (e.g., mutations in two‐component regulatory systems) can trigger such porin and efflux alterations under antibiotic stress [41]. Overall, the mechanistic landscape in India is one of *NDM-centered resistance*, often accompanied by OXA‐type enzymes and bolstered by porin/efflux changes—a formidable combination.

#### 3.2.4. Additional Mechanisms of Carbapenem Resistance

In addition to carbapenemase production, carbapenem resistance may arise through several alternative mechanisms. ESBLs or AmpC *β*‐lactamases, when combined with reduced porin expression, can confer clinically significant resistance, particularly in *K. pneumoniae* and *E. coli* [33, 35, 42]. Alterations in penicillin‐binding proteins may reduce carbapenem binding, especially in nonfermenters [18]. Biofilm formation by organisms such as *P. aeruginosa* and *A. baumannii* limits antibiotic penetration and promotes tolerant dormant cells [18]. Mutations in regulatory pathways (e.g., PhoPQ, PmrAB) can modify membrane permeability and stress responses, indirectly enhancing resistance [43]. In addition, heteroresistance, where small resistant subpopulations coexist within apparently susceptible isolates, and persister‐cell mediated adaptive tolerance may contribute to treatment failure and recurrent infection.

### 3.3. Clinical Impact and Outcomes

Patients with carbapenem‐resistant infections in India tend to have worse clinical outcomes compared to those with carbapenem‐susceptible infections. Several studies in our review documented significantly higher mortality rates, longer hospital stays, and increased costs associated with CRE and carbapenem‐resistant *A. baumannii and P.* aeruginosa cases [9, 15, 44]. For instance, as noted earlier, bloodstream infections by carbapenem‐resistant *K. pneumoniae* were associated with mortality approaching 50%–70% in some ICU cohorts [4]. Even outside of critical care, CRE bacteremia has a case fatality rate roughly double that of non‐CRE bacteremia (often exceeding 40% within 30 days) [14, 21]. Invasive infections by carbapenem‐resistant *A. baumannii* (such as ventilator‐associated pneumonia) are notoriously difficult to treat and also carry very high mortality [9], frequently > 50% in ventilated patients, according to reports. Beyond mortality, these infections often require prolonged hospitalization and may necessitate toxic salvage antimicrobial therapies (like polymyxins), which contribute to organ damage and other complications [45, 46]. The review also highlighted the challenge of delayed effective antimicrobial therapy; many patients initially receive ineffective empiric antibiotics (since resistance is not known immediately), and each hour of delay in appropriate treatment increases the risk of adverse outcome [15]. This underscores the importance of rapid diagnosis and tailored therapy (addressed in Section 4). Finally, epidemiologic studies indicated that patients with prior healthcare exposures—such as recent surgery, ICU admission, or prior broad‐spectrum antibiotic use—were at highest risk of carbapenem‐resistant infections [21, 30], suggesting that improved stewardship and infection control could mitigate some of the burden. Table S1 gives a summary of all the 75 articles included in this review.

## 4. Discussion

### 4.1. Key Findings and Comparison to Global Data

Our systematic review confirms that carbapenem resistance in India has escalated to critical levels in the period 2019–2024. The pooled evidence aligns with global observations that South Asia is a hotspot for carbapenem‐resistant infections [2]. In India, roughly one‐third to one‐half of gram‐negative bacterial hospital infections are now caused by organisms nonsusceptible to carbapenems, which is an extremely high proportion by international standards [14, 15]. The situation in India is comparable to that in neighboring South Asian countries (e.g., Pakistan, Bangladesh) where NDM‐driven resistance also predominates [6]. It contrasts with regions like North America or Europe, where carbapenemase‐producing *K. pneumoniae* (often KPC enzyme) is present but at lower overall prevalence and where aggressive infection control has contained spread in many hospitals. Notably, the dominance of NDM and OXA‐48‐like enzymes in India differs from the KPC‐centric epidemiology seen in the United States [47, 48]. However, the end result is similar—conventional antibiotics becoming largely ineffective. Another finding from the review is the diversity of resistance mechanisms coexisting in Indian isolates (multiple genes, plasmids, etc.) [28, 36, 39], reflecting a decade of accumulation of resistance elements since NDM‐1′s emergence. This diversity could complicate molecular surveillance (since one must screen for several genes) and also means that any single new antibiotic may not suffice as a solution.

The devastating clinical outcomes associated with these infections in India (50% + mortality in serious cases) echo global reports and emphasize that carbapenem‐resistant infections are a true emergency [9, 44]. A 2025 global analysis of bloodstream infections highlighted carbapenem‐resistant *K. pneumoniae*, *A. baumannii*, and *P. aeruginosa* as leading causes of death in bacteremic patients [2]. Our review finds the same pathogens at the forefront in Indian hospitals [9, 16, 18]. This synergy of global and local data reinforces the call for urgent interventions in India—including novel therapeutics, strengthened antimicrobial stewardship, and improved public health strategies—to curb the tide of carbapenem resistance.

The diagnostic and therapeutic stewardship strategies proposed in this review are directly informed by the observed gaps in diagnostic utilization and variability in treatment approaches identified across included studies.

### 4.2. Diagnostic Challenges and Stewardship

Early diagnosis of carbapenem‐resistant infections is crucial for patient management and infection control, yet it remains challenging in many Indian settings. Most laboratories in India rely on conventional antibiotic susceptibility testing to flag possible carbapenem resistance (e.g., elevated MICs to imipenem/meropenem). Confirming the mechanism (carbapenemase production) often requires additional tests that may not be widely available. Phenotypic assays like the Modified Hodge Test (MHT), Carba NP test, or the Modified Carbapenem Inactivation Method (mCIM) with EDTA (to detect MBLs) are used in some centers [5, 33, 43]. These tests are relatively inexpensive and can indicate the presence of a carbapenemase (and sometimes its class), but they have limitations in sensitivity and turn‐around time. Newer rapid molecular tests (e.g., PCR panels or immunochromatographic assays) can directly identify common carbapenemase genes (NDM, OXA‐48, KPC, etc.) within hours, greatly aiding targeted therapy. However, cost and technical expertise limit deployment of molecular diagnostics in many resource‐constrained Indian hospitals [5, 23]. Our review noted that only a minority of studies performed gene sequencing; most inferred mechanisms from phenotypic patterns. There is a clear need to expand rapid diagnostic capabilities for CRE in India—for example, implementing LAMP assays or automated PCR cartridges in tertiary centers [5]—to guide timely treatment. In the interim, clinicians must maintain a high index of suspicion for CRE in high‐risk patients and institute empiric precautions (e.g., contact isolation) and broad‐spectrum coverage of antimicrobial therapy when appropriate, even before laboratory confirmation [49].

Antimicrobial stewardship programs (AMSPs) are essential to slow the spread of resistance. The period 2019–2024 saw increasing efforts in some Indian hospitals to restrict carbapenem use and promote alternatives when feasible [50, 51]. Nevertheless, inappropriate antibiotic use remains a major driver of resistance. Fluoroquinolones and third‐generation cephalosporins (often overused in the community) can predispose patients to CRE colonization/infection [48]. Limiting unnecessary use of these and carbapenems, and de‐escalating therapy based on culture results, are key stewardship strategies [24, 50]. The review findings support continued education and guidelines to optimize antibiotic prescribing. Additionally, rigorous infection control measures (screening for CRE colonization, hand hygiene, equipment disinfection, and cohorting of colonized patients) have been recommended by the WHO and National guidelines [1]. These measures need better implementation across Indian healthcare facilities to prevent hospital outbreaks of CRE and other multidrug‐resistant organisms [30, 39].

### 4.3. Clinical Guidance for Diagnosis and Management

Translating the evidence from this review into clinical practice, we provide an updated clinical flowchart and recommendations for managing carbapenem‐resistant infections in India. The guidance below synthesizes current international and Indian expert guidelines with the findings of our review:

#### 4.3.1. Early Recognition and Diagnostic Workup

**Figure 2:**
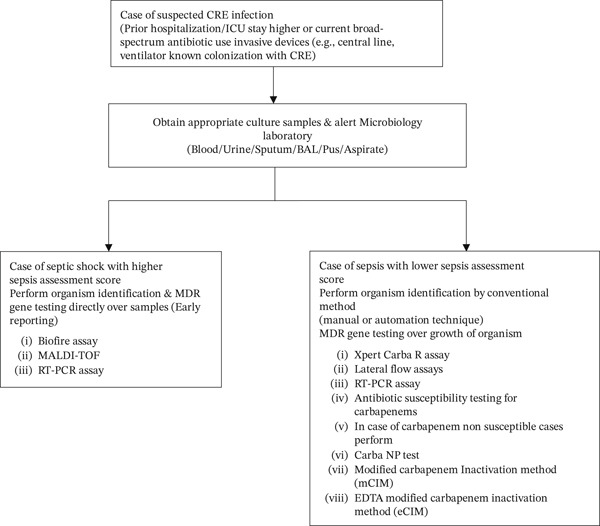
Diagnostic flowchart for suspected carbapenem‐resistant infection.

Figure 2 presents a flowchart for the early recognition and diagnostic approach to suspected CRE infections. Key steps include as follows:

- Risk factor assessment: Patients with prior carbapenem use, prolonged hospitalization/ICU stay, invasive devices, or known colonization with CRE should be considered at high risk [4, 21, 52]. If such a patient presents with signs of serious gram‐negative infection (e.g., sepsis, pneumonia), clinicians should suspect carbapenem‐resistant pathogens from the outset.
- Empiric isolation and precautions: Initiate contact precautions (gloves, gowns, possibly cohorting) if CRE is suspected, to prevent spread [1, 30].
- Obtain cultures and alert microbiology laboratory: Send appropriate cultures (blood, urine, sputum, etc.) before starting antibiotics. Alert the microbiology laboratory if CRE is suspected, so that they perform carbapenemase screening tests in parallel with routine susceptibility. If available, request a rapid carbapenemase gene test (e.g., GeneXpert Carba‐R or equivalent) on critical samples [5, 35]—this can confirm the presence of NDM, OXA‐48, etc., within hours or in few minutes
- Phenotypic confirmation: If molecular tests are not available, phenotypic carbapenemase detection must be done on isolates with reduced carbapenem susceptibility. Recommended methods as per CLSI guidelines include mCIM/eCIM (EDTA‐modified CIM; to differentiate MBLs) or the Carba NP test [2, 43, 48]. These will guide subsequent therapy choices by identifying the carbapenemase class (metallo‐*β*‐lactamase vs. serine carbapenemase). Among the included studies, diagnostic approaches varied considerably. Conventional phenotypic methods, such as disc diffusion and broth microdilution were universally used for susceptibility testing. However, only a subset of studies (approximately 38%) incorporated molecular diagnostics such as PCR‐based detection of carbapenemase genes (e.g., *blaNDM*, *blaOXA-48*) [5, 32].
- Phenotypic confirmatory tests including mCIM, eCIM, and Carba NP test were reported in several studies [33, 43], though their adoption remained inconsistent [32].
- Advanced diagnostic modalities such as MALDI‐TOF and rapid molecular assays were limited to tertiary care and research settings [23, 38], highlighting a significant gap in diagnostic capacity across India.

Figure 2 gives a diagnostic algorithm that emphasizes not waiting for full AST results if the patient is very ill—early broad‐spectrum treatment should be started if CRE is likely, then adjusted when lab results return.

#### 4.3.2. Empiric Therapy for Suspected CRE

**Figure 3:**
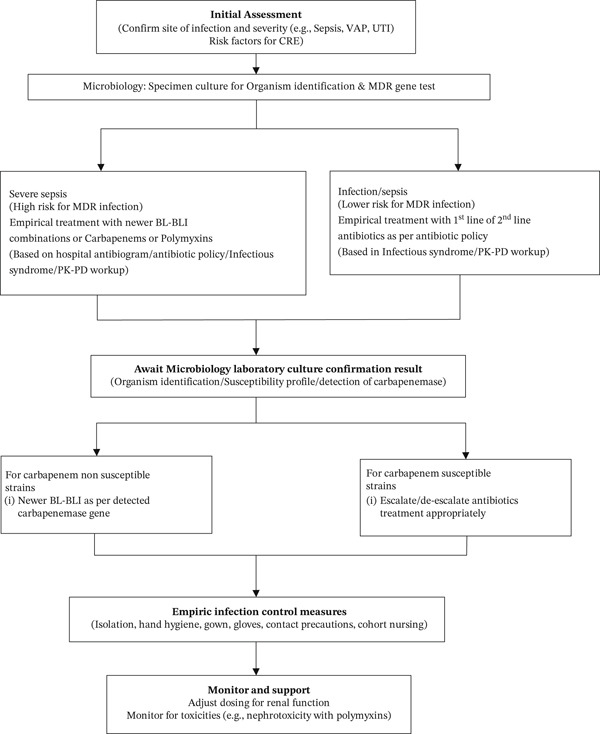
Management algorithm for carbapenem‐resistant gram‐negative infections.

When a high‐risk patient has a severe infection and CRE is suspected, empiric therapy should cover the possibility of a resistant organism. International guidelines (e.g., IDSA 2023) recommend avoiding carbapenem monotherapy in such cases because it will likely fail [48]. Instead, a combination regimen or a newer agent effective against CRE should be used empirically. Based on our review and current practice:

- For suspected CRE infections, empiric options include an advanced *β*‐lactam/*β*‐lactamase inhibitor if available (see next section), often in combination with a second agent. For example, one empiric approach in a critically ill patient is ceftazidime‐avibactam (CAZ‐AVI) plus amikacin (an aminoglycoside)—this covers many CRE pending identification [45, 53]. Alternatively, colistin (polymyxin E) combined with a carbapenem (for possible additive effect) has been used, though toxicity is a concern [11, 45, 46, 48]. Local microbiology patterns matter; hospitals with access to newer drugs may use them upfront.
- For suspected carbapenem‐resistant *A. baumanni* infections (e.g., ventilator‐associated pneumonia), colistin‐based therapy is often the only viable empiric option in many Indian ICUs, sometimes combined with high‐dose sulbactam (if *A. baumannii* is sulbactam susceptible) or tigecycline [16, 31]. Cefiderocol can be a potent empiric drug against *A. baumannii,* if available [9, 12, 16].
- In all cases, empiric regimens should be streamlined once susceptibility results are known. Figure 3 (management algorithm) illustrates the step‐by‐step approach: Start broad if CRE is suspected in a critically ill patient, then narrow therapy based on confirmed organism and mechanism [11, 49].

#### 4.3.3. Definitive Therapy Based on Carbapenemase Type

Optimal treatment of a confirmed carbapenem‐resistant infection depends on the organism′s carbapenemase production and overall susceptibility profile. The following guidance is aligned with the Indian Council of Medical Research (ICMR) 2022 national recommendations and recent expert consensus: [11, 49].

- NDM or other MBL‐producer (Metallo‐*β*‐lactamase): These enzymes (e.g., NDM‐1) hydrolyze almost all *β*‐lactams except aztreonam. However, MBL‐producers often also carry ESBLs or AmpC that inactivate aztreonam unless protected. Recommended therapy is CAZ‐AVI plus aztreonam in combination [11]. CAZ‐AVI by itself does not inhibit MBLs like NDM, but it can protect aztreonam from any co‐produced serine *β*‐lactamases, while aztreonam is stable to the MBL. This synergistic combination has been endorsed by ICMR and international guidelines as the most effective option for NDM‐producing CRE [49, 53]. If CAZ‐AVI is not available, alternatives are limited: colistin in combination with meropenem (high‐dose, prolonged infusion) or with tigecycline has been used, but with lower success [46, 54]. Cefiderocol, a novel siderophore cephalosporin, is another option active against MBL‐producing Enterobacterales (see following sections) [12].
- OXA‐48‐like producer: Carbapenem‐resistant isolates with an OXA‐48 or related enzyme (and no NDM) are usually best treated with CAZ‐AVI monotherapy [49]. Evidence indicates OXA‐48‐like *K. pneumoniae* infections respond well to CAZ‐AVI, which inhibits these Class D enzymes effectively. The ICMR 2022 guidelines specifically recommend CAZ‐AVI as first‐line for OXA‐48 producers [49]. In instances where avibactam is not accessible, meropenem‐vaborbactam (another newer carbapenem–inhibitor combination) could be effective if the isolate is OXA‐48 (meropenem‐vaborbactam is more tailored to KPC though). Older drugs like colistin or tigecycline are less preferred here given CAZ‐AVI’s high efficacy [45, 46].
- KPC producer: Although KPC‐type carbapenemases are infrequent in India [28, 52], they may be encountered, especially in patients with healthcare exposure abroad. These are best treated with meropenem‐vaborbactam (a beta‐lactamase inhibitor targeting KPC) or with CAZ‐AVI [11, 48]. Both regimens have shown good outcomes in KPC‐CRE infections. If neither is available, polymyxin‐based combination therapy or tigecycline can be considered, but with caution due to inferior efficacy.
- Noncarbapenemase CRE: A subset of CRE (especially some *E. coli*) is carbapenem‐resistant by mechanisms like ESBL plus porin loss, without a carbapenemase gene [32, 33, 35]. If lab tests confirm the absence of carbapenemase, high‐dose carbapenems (e.g., meropenem in extended infusion) may still be useful [48, 49]. For serious infections, however, many clinicians would still opt for a drug like CAZ‐AVI if the isolate is susceptible, to ensure adequate coverage [54]. It is important to tailor therapy to susceptibility results. Most of the newer agents require testing via reference or specialized methods. Clinicians should work closely with microbiologists to interpret MICs for drugs like colistin, tigecycline, and newer combos, as breakpoints and testing methods vary [45, 48].

#### 4.3.4. New and Emerging Therapies

The landscape of CRE treatment has improved somewhat with the advent of new antimicrobial agents in recent years. Our review touched on some emerging options, and we briefly highlight them in context:

- CAZ‐AVI: As noted, CAZ‐AVI has become a cornerstone for treating CRE in India, particularly infections caused by serine carbapenemase‐producing organisms [11, 49]. It is most effective against isolates producing OXA‐48‐like carbapenemases (commonly prevalent in India) and KPC enzymes, as avibactam inhibits these *β*‐lactamases [25, 37]. CAZ‐AVI also retains activity against some CRE strains in which resistance is mediated by ESBL or AmpC production combined with porin loss, provided no metallo‐*β*‐lactamase is present [35]. However, CAZ‐AVI alone is not active against metallo‐*β*‐lactamase (MBL) producers, such as NDM, VIM, or IMP, because avibactam does not inhibit these enzymes. For this reason, in regions like India where NDM‐producing CRE are common, CAZ‐AVI is often used in combination with aztreonam, which can bypass MBL hydrolysis while avibactam protects aztreonam from coproduced ESBLs or OXA‐48 enzymes [11, 49] . It was introduced in India around 2019 and has since been increasingly adopted [53]. Clinical studies have shown superior outcomes with CAZ‐AVI–based regimens compared with older polymyxin‐based therapy for CRE infections [45, 53]. One important caveat is the need for proper dosing in renal impairment; under‐dosing CAZ‐AVI in such patients has been associated with higher treatment failure and the emergence of resistance during therapy [45].
- Meropenem‐vaborbactam and imipenem‐cilastatin‐relebactam: These newer combinations target KPC and some other Class A/C enzymes. Athough not yet widely available in India, they could play a role if KPC‐mediated CRE increases. Meropenem‐vaborbactam has shown excellent outcomes against KPC‐CRE in trials, and relebactam expands imipenem′s activity to include certain resistant *Pseudomonas*. Their utility against OXA‐48 or NDM producers is limited (ineffective against NDM [48, 54], and only partial against OXA‐48), so they are niche options in India at present.
- Cefiderocol: This novel siderophore cephalosporin, active against a broad range of carbapenem‐resistant gram‐negatives, including MBL‐producers and *A. baumannii*, is a promising agent for India′s CRE crisis. A pivotal trial (CREDIBLE‐CR) demonstrated that cefiderocol can be as effective as best available therapy (and often with lower nephrotoxicity than colistin) in serious infections caused by carbapenem‐resistant bacteria [12]. Cefiderocol is particularly valuable for NDM‐producing CRE and carbapenem‐resistant *Acinetobacter*, as it can overcome metallo‐*β*‐lactamases and has potent activity against *A. baumannii* [16, 31]. As of 2025, access to cefiderocol in India is limited and its cost is high [9, 45], but it may become an important tool in tertiary centers for the most resistant infections.
- Plazomicin: A next‐generation aminoglycoside, plazomicin, has enhanced activity against certain CRE (including some enzymatic resistance bypass). It iss mainly considered for complicated UTIs due to CRE. Not yet widely used in India [48], but it could be considered if available for UTI caused by CRE that are resistant to other orals.
- Combinations and others: Various double or triple combinations (e.g., colistin plus high‐dose carbapenem plus tigecycline) have been tried in desperation for panresistant infections [46, 54]. Outcomes are variable and toxicity is significant. The consensus in recent literature is that monotherapy with a highly active new agent (like CAZ‐AVI or cefiderocol) is preferred, when possible, rather than cobbling together multiple older drugs [11]. Combination therapy may still be used empirically or in severe infections to ensure coverage until susceptibilities confirm the best single agent.

Other therapeutic options for CRE infections include polymyxins (colistin or polymyxin B), which were previously widely used in India but are now reserved mainly for salvage therapy because of nephrotoxicity and inferior outcomes compared with newer agents [45, 46]. Tigecycline remains useful for complicated intra‐abdominal, soft tissue, and polymicrobial infections, although its low serum and urinary levels limit use in bacteremia and urinary tract infections. Fosfomycin is particularly valuable for urinary tract infections and as part of combination therapy due to its unique mechanism of action and favorable safety profile [43]. Aminoglycosides such as amikacin may still be effective against selected susceptible isolates, especially in urinary infections [45, 53], but their nephrotoxicity and ototoxicity restrict prolonged use. Newer or less widely available agents such as eravacycline, plazomicin, temocillin, and cefiderocol may further expand treatment options, particularly for highly resistant isolates, although access and cost remain limiting factors in India [9, 12].

#### 4.3.5. Indian Expert Consensus (ICONIC‐II 2024)

It is worth noting that a panel of Indian experts convened in 2023–24 (ICONIC‐II) to formulate consensus statements on managing CRE in critically ill patients [11]. The consensus emphasizes an individualized approach: “prompt, tailored empiric therapy and combination therapies based on local epidemiology.” They advocate for active surveillance of CRE colonization in hospitals [21, 52], early use of CAZ‐AVI (with aztreonam when MBLs are present) as the preferred therapy for most CRE, and judicious use of older drugs like colistin (reserved for when no better options exist). The panel′s treatment algorithm (Figure 3) suggests initial combination therapy in critically ill patients suspected of CRE, covering both Enterobacterales and nonfermenters, followed by de‐escalation once the specific pathogen and its carbapenemase type are known. Our review′s findings support these recommendations: the highest survival rates were seen when appropriate effective therapy (often a new *β*‐lactam/*β*‐lactamase inhibitor) was started early [15, 53]. Conversely, delays or reliance on older agents like colistin were linked to poorer outcomes [9, 46].

### 4.4. Limitations

This review has several limitations. A formal meta‐analysis was not performed due to substantial heterogeneity in study design, populations, diagnostic methods, and outcome reporting, limiting quantitative synthesis. Most included studies were single‐center with variable methodological quality, restricting precise estimation of pooled resistance rates. Publication bias is possible, as data from tertiary care centers may be overrepresented. Variability in diagnostic techniques may have led to misclassification of resistance mechanisms. Additionally, inconsistent reporting of clinical outcomes limited robust assessment of treatment effectiveness. Finally, studies published after December 2024 were not included.

Despite these limitations, consistent findings across diverse settings support the overall robustness of the conclusions.

### 4.5. Future Directions

Our review highlights an urgent need for strengthened surveillance of carbapenem resistance in India. A coordinated national database (integrating the AMR surveillance network reports [49]) would help track trends and the emergence of novel resistance genes (like future NDM variants or transferable OXA) [27, 36]. On the research front, genomic studies of CRE in India [10, 11, 17, 27] could illuminate transmission patterns and clonal lineages, informing infection control strategies. From a clinical standpoint, rapid diagnostic tools and increased availability of newer antimicrobial agents are paramount. Hospitals should invest in rapid CRE detection (e.g., molecular assays or MALDI‐TOF‐based carbapenemase detection) to guide therapy [23, 38]. Policymakers and healthcare leaders in India must also address antibiotic overuse through stricter regulations and public education about antibiotic stewardship [24, 36, 50, 51]. Finally, infection prevention measures—hand hygiene, cleaning protocols, antibiotic stewardship—are the front‐line defenses and need more robust implementation across both public and private healthcare sectors [30, 50].

## 5. Conclusion

Carbapenem resistance has established itself as a formidable challenge in India′s healthcare landscape, with CRE and other carbapenem‐resistant pathogens becoming increasingly common causes of severe infections from 2019 to 2024. This systematic review demonstrates that high rates of resistance (often 30%–60%) now prevail in *K pneumoniae*, *E. coli*, and *A.baumannii*, and *Pseudomonas* spp. isolates in many Indian hospitals [14, 15, 19, 20, 44]. The NDM‐1 metalloenzyme and its kin continue to drive most of this resistance [28, 35, 37], frequently accompanied by OXA‐48‐like enzymes [25, 36, 39], making Indian CRE some of the most drug‐resistant in the world. The clinical repercussions—high mortality, limited treatment options [9, 44]—underscore the urgent need for action. On a hopeful note, emerging therapies like CAZ‐AVI (± aztreonam) and cefiderocol [12, 53] offer improved outcomes and are gradually changing the treatment paradigm for CRE infections in India, as reflected in recent guideline updates.

Going forward, a multipronged strategy is required: (1) enforce robust infection control and antibiotic stewardship to prevent spread and emergence of resistance [24, 50, 51]; (2) enhance laboratory capacity for rapid detection of carbapenemases [23, 38]; (3) ensure new effective drugs are accessible and used judiciously [12, 45, 53]; and (4) continue surveillance and research into resistance mechanisms [6, 10, 11, 27]. With concerted effort, it is possible to curb the impact of carbapenem‐resistant superbugs. The clinical guidance and flowcharts provided in this review serve as a practical tool for physicians, aiding in early recognition and optimal management of CRE infections. Tackling carbapenem resistance in India will ultimately require sustained collaboration between clinicians, microbiologists, public health authorities, and policy‐makers—an effort as critical as any in modern medicine.

## Author Contributions

Akhil Kapoor conceived the study, designed the review, and drafted the manuscript. Akhil Kapoor, Madhu Singh, Rahul Sarode, and Vijeta Bajpai performed the literature search, data extraction, and quality assessment. Anuj Gupta, Bipinesh Sansar, Sujit Bharti, Anwita Mishra, Anil Singh, and Ankita Chaurasia contributed to the interpretation of findings and manuscript revision.

## Funding

No funding was received for this manuscript.

## Disclosure

Artificial intelligence tools were used solely for language refinement and editorial assistance. All scientific content, interpretation, and conclusions were independently developed and verified by the authors. All authors read and approved the final version of the manuscript.

## Ethics Statement

This study is a systematic review of previously published articles. Ethics approval and consent to participate were not required.

## Consent

The authors have nothing to report.

## Conflicts of Interest

The authors declare no conflicts of interest.

## Supporting information


**Supporting Information** Additional supporting information can be found online in the Supporting Information section. Table S1: PRISMA Checklist. Additional references that are not cited within the main text have been provided in the supporting file. These references were reviewed during the literature assessment process and contributed to identifying research gaps, refining the problem statement, and developing the broader analytical insights presented in this review.

## Data Availability

All data generated or analyzed during this review are included in this published article and its supporting information files. Additional datasets are available from the corresponding author on reasonable request.

## References

[1] World Health Organization , Guidelines for the Prevention and Control of Carbapenem-Resistant Enterobacteriaceae, Acinetobacter baumannii and Pseudomonas aeruginosa in Health Care Facilities, 2017, World Health Organization. 29630191

[2] Antimicrobial Resistance Collaborators , Global Burden of Bacterial Antimicrobial Resistance in 2019: A Systematic Analysis, Lancet. (2022) 399, no. 10325, 629–655, 10.1016/S0140-6736(21)02724-03. 35065702 PMC8841637

[3] Kumarasamy K. K. , Toleman M. A. , Walsh T. R. , Bagaria J. , Butt F. , Balakrishnan R. , Chaudhary U. , Doumith M. , Giske C. G. , Irfan S. , Krishnan P. , Kumar A. V. , Maharjan S. , Mushtaq S. , Noorie T. , Paterson D. L. , Pearson A. , Perry C. , Pike R. , Rao B. , Ray U. , Sarma J. B. , Sharma M. , Sheridan E. , Thirunarayan M. A. , Turton J. , Upadhyay S. , Warner M. , Welfare W. , Livermore D. M. , and Woodford N. , Emergence of a New Antibiotic Resistance Mechanism in India, Pakistan, and the UK: a molecular, biological, and epidemiological study, Lancet Infectious Diseases. (2010) 10, no. 9, 597–602, 10.1016/S1473-3099(10)70143-2, 20705517. 20705517 PMC2933358

[4] Veeraraghavan B. , Shankar C. , Karunasree S. , Kumari S. , Ravi R. , and Ralph R. , Carbapenem Resistant Klebsiella pneumoniae isolated From Bloodstream Infection: Indian Experience, Pathogens and Global Health. (2017) 111, no. 5, 240–246, 10.1080/20477724.2017.1340128. 28670975 PMC5560201

[5] Verma G. , Nayak S. R. , Jena S. , Panda S. S. , Pattnaik D. , Praharaj A. K. , and Singh N. , Prevalence of carbapenem-resistant Enterobacterales, Acinetobacter baumannii, and Pseudomonas aeruginosa in a tertiary care hospital in eastern India: a pilot study, Journal of Pure and Applied Microbiology. (2023) 17, no. 4, 2243–2249, 10.22207/JPAM.17.4.21.

[6] Das S. , The crisis of carbapenemase-mediated carbapenem resistance across the human–animal–environmental interface in India, Infectious Diseases Now. (2023) 53, no. 1, 104628, 10.1016/j.idnow.2022.09.023. 36241158

[7] Dixit A. , Kumar N. , Kumar S. , and Trigun V. , Antimicrobial resistance: progress in the decade since emergence of New Delhi metallo-*β*-lactamase in India, Indian Journal of Community Medicine. (2019) 44, no. 1, 4–8, 10.4103/ijcm.IJCM_217_18. 30983704 PMC6437806

[8] Kunjalwar R. and Mudey G. , A cross sectional study on endemicity of VIM, NDM, KPC, IPM & OXA-48 genes in Carbapenemase producing Klebsiella pneumoniae and Escherichia coli from a tertiary hospital using mCIM, eCIM, and PCR in central India, F1000Research. (2024) 13, 10.12688/f1000research.147644.1.

[9] Das B. J. , Banerjee T. , Wangkheimayum J. , Mishra K. , Kumar A. , and Bhattacharjee A. , Characterisation of blaOXA-232 carrying carbapenem-resistant Klebsiella pneumoniae (CRKP) & their expression profiles under selective carbapenem pressure: An in-depth study from India, Indian Journal of Medical Research. (2024) 159, no. 6, 644–652, 10.25259/IJMR_1915_22. 39382472 PMC11463862

[10] Prayag P. S. , Patwardhan S. A. , Joshi R. S. , Panchakshari S. P. , Rane T. , and Prayag A. P. , Enzyme patterns and factors associated with mortality among patients with carbapenem resistant Acinetobacter baumannii (CRAB) bacteremia: real world evidence from a tertiary center in India, Indian Journal of Critical Care Medicine. (2023) 27, no. 9, 10.5005/jp-journals-10071-24534. PMC10504652 37719354

[11] Nagaraj G. , Shamanna V. , Govindan V. , Rose S. , Sravani D. , Akshata K. P. , Shincy M. R. , Venkatesha V. T. , Abrudan M. , Argimón S. , Kekre M. , Underwood A. , Aanensen D. M. , Ravikumar K. L. , NIHR Global Health Research Unit on Genomic Surveillance of Antimicrobial Resistance , Abudahab K. , Harste H. , Muddyman D. , Taylor B. , Wheeler N. , David S. , Donado-Godoy P. , Bernal J. F. , Arevalo A. , Valencia M. F. , Osma Castro E. C. D. , Ravishankar K. N. , Okeke I. N. , Oaikhena A. O. , Afolayan A. O. , Ajiboye J. J. , Odih E. E. , Carlos C. , Lagrada M. L. , Macaranas P. K. V. , Olorosa A. M. , Gayeta J. M. , and Herrera E. M. , High-resolution genomic profiling of carbapenem-resistant Klebsiella pneumoniae isolates: a multicentric retrospective Indian study, Clinical Infectious Diseases. (2021) 73, no. supplement 4, S300–S307, 10.1093/cid/ciab767, 34850832. 34850832 PMC8634558

[12] Indian Council of Medical Research (ICMR). Diagnosis & Management of Carbapenem-Resistant Gram-Negative Infections: Guidance Document, 2022, ICMR.

[13] Bassetti M. , Echols R. , Matsunaga Y. , Ariyasu M. , Doi Y. , Ferrer R. , Lodise T. P. , Naas T. , Niki Y. , Paterson D. L. , Portsmouth S. , Torre-Cisneros J. , Toyoizumi K. , Wunderink R. G. , and Nagata T. D. , Efficacy and safety of cefiderocol or best available therapy for the treatment of serious infections caused by carbapenem-resistant Gram-negative bacteria (CREDIBLE-CR): a randomised, open-label, multicentre, pathogen-focused, descriptive, phase 3 trial, Lancet Infectious Diseases. (2021) 21, no. 2, 226–240, 10.1016/S1473-3099(20)30796-9. 33058795

[14] Indrajith S. , Mukhopadhyay A. K. , Chowdhury G. , Farraj D. A. A. , Alkufeidy R. M. , Natesan S. , Meghanathan V. , Gopal S. , and Muthupandian S. , Molecular Insights of Carbapenem Resistance Klebsiella pneumoniae Isolates With Focus on Multidrug Resistance From Clinical Samples, Journal of Infection and Public Health. (2021) 14, no. 1, 131–138, 10.1016/j.jiph.2020.09.018, 33234410. 33234410

[15] Kaur J. , Singh H. , and Sethi T. , Emerging Trends in Antimicrobial Resistance in Bloodstream Infections: Multicentric Longitudinal Study in India (2017–2022), Lancet Regional Health-Southeast Asia. (2024) 26, 100412, 10.1016/j.lansea.2024.100412. 38757091 PMC11097075

[16] Sathyanarayana P. , Mannur Y. S. , Mudigubba M. K. , and Lingaiah M. , Emerging challenges and innovations in the management of carbapenem-resistant Enterobacteriaceae infections in hospital settings, Anti-Infect Agents. Published online November. (2024) 12, 10.2174/0122113525347025241015053800.

[17] Paneri M. , Sevta P. , and Yagnik V. D. , Burden of Carbapenem ResistantAcinetobacter baumanniiHarboringblaOXAGenes in the Indian Intensive Care Unit, Global Journal of Medical Pharmaceutical & Biomedical Update. (2023) 18, 10.25259/GJMPBU_18_2023.

[18] Bansal K. , Saroha T. , Patil P. P. , Kumar S. , Kumar S. , Singhal L. , Gautam V. , and Patil P. B. , Evolutionary Trends of Carbapenem-Resistant and Susceptible Acinetobacter baumannii Isolates in a Major Tertiary Care Setting From North India, Infection, Genetics and Evolution. (2024) 117, 105542, 10.1016/j.meegid.2023.105542. 38122920

[19] Menon N. D. , Somanath P. , Jossart J. , Vijayakumar G. , Shetty K. , Baswe M. , Chatterjee M. , Hari M. B. , Nair S. , Kumar V. A. , Nair B. G. , Nizet V. , Perry J. J. P. , and Kumar G. B. , Comparative Molecular Profiling of Multidrug-Resistant Pseudomonas aeruginosa Identifies Novel Mutations in Regional Clinical Isolates From South India, JAC-Antimicrobial Resistance. (2024) 6, no. 1, 10.1093/jacamr/dlae001, 38230352. PMC10789591 38230352

[20] Prabhala S. , Sundaresan A. , and Varaiya A. , Prevalence and Molecular Characterization of Carbapenem Resistant Gram-Negative Bacilli in a Tertiary Care Hospital in Mumbai, IP International Journal of Medical Microbiology and Tropical Diseases. (2023) 9, 150–154, 10.18231/j.ijmmtd.2023.030.

[21] Sharma K. , Tak V. , Nag V. L. , Bhatia P. K. , and Kothari N. , An Observational Study on Carbapenem-Resistant Enterobacterales (CRE) Colonisation and Subsequent Risk of Infection in an Adult Intensive Care Unit at a Tertiary Care Hospital in India, Infection Prevention in Practice. (2023) 5, no. 4, 100312, 10.1016/j.infpip.2023.100312. 37868258 PMC10585280

[22] Premanadham N. , Munilaxmi S. K. , and Avinash R. K. , Prevalence of Carbapenem-Resistant Enterobacteriaceae Isolated in Tertiary Care Hospital in NMC and Hospital, Nellore, Andhra Pradesh, India, International Journal of Current Microbiology and Applied Sciences. (2022) 11, no. 4, 10.20546/ijcmas.2022.1104.017.

[23] Rajni E. , Duggal S. , Gajjar D. , Sharma R. , Garg V. , and Khatri P. K. , First Report of Molecular Epidemiology of Carbapenem-Resistant Enterobacteriaceae From a Tertiary Level Hospital in Rajasthan, Western India, Journal of Medical Sciences. (2022) 8, no. 3, 10.46347/jmsh.v8i3.22.88.

[24] Lahiry S. , Ramalingam R. , Dalal K. , Pawar A. , Chugh Y. , Gandhi A. , and Kotak B. , Tackling AMR Crisis in India: Changing Paradigm, Asian Journal of Medical Sciences. (2020) 11, no. 6, 129–137, 10.3126/ajms.v11i6.29427.

[25] Joshi D. N. , Shenoy B. , Bhavana M. V. , Adhikary R. , Shamarao S. , and Mahalingam A. , Prevalence of Carbapenem-Resistant Enterobacteriaceae and the Genes Responsible for Carbapenemase Production in a Tertiary Care Hospital in South India, Emergency Medicine Journal. (2023) 12, no. 3, 462–467, 10.33590/emj/1030042.

[26] Jaggi N. , Chatterjee N. , Singh V. , Giri S. K. , Dwivedi P. , Panwar R. , and Sharma A. P. , Carbapenem Resistance in Escherichia coli and Klebsiella pneumoniae Among Indian and International Patients in North India, Acta microbiologica et immunologica Hungarica. (2019) 66, no. 3, 367–376, 10.1556/030.66.2019.020, 31438725. 31438725

[27] Halder G. , Chaudhury B. N. , Mandal S. , Denny P. , Sarkar D. , Chakraborty M. , Khan U. R. , Sarkar S. , Biswas B. , Chakraborty A. , Maiti S. , and Dutta S. , Whole genome Sequence-Based Molecular Characterization of Blood Isolates of Carbapenem-Resistant Enterobacter cloacae Complex From ICU Patients in Kolkata, India, During 2017–2022: Emergence of Phylogenetically Heterogeneous Enterobacter hormaechei Subsp. xiangfangensis, Microbiology Spectrum. (2024) 12, no. 4, e03523–e03529, 10.1128/spectrum.03529-23, 38385742. PMC10986559 38385742

[28] Kumari N. , Kumar M. , Katiyar A. , Kumar A. , Priya P. , Kumar B. , Biswas N. R. , and Kaur P. , Genome-Wide Identification of Carbapenem-Resistant Gram-Negative Bacterial (CR-GNB) Isolates Retrieved From Hospitalized Patients in Bihar, India, Scientific Reports. (2022) 12, no. 1, 10.1038/s41598-022-12471-3, 35590022. PMC9120164 35590022

[29] Ralte V. S. , Loganathan A. , Manohar P. , Sailo C. V. , Sanga Z. , Ralte L. , Zothanzama J. , Leptihn S. , Nachimuthu R. , and Kumar N. S. , The Emergence of Carbapenem-Resistant Gram-Negative Bacteria in Mizoram, Northeast India, Microbiology Research. (2022) 13, no. 3, 342–349, 10.3390/microbiolres13030027.

[30] Fallon S. , Agrawal U. , Basu S. , Fabre V. , Fisseha S. , Johns Hopkins Hospital , Kakrani A. , Karyakarte R. , Kumar M. , Mane A. , Mave V. , Mehta Y. , Mirza S. , Patel A. , Randive B. , Rathod P. , Robinson M. , Rodrigues C. , Sarma S. , Shah J. , Simner P. , Singh S. , and Curless M. , Antimicrobial Stewardship and Healthcare Epidemiology, 2024, 4, no. S1, S104–S105, 10.1017/ash.2024.252.

[31] Seshatri S. , Charan J. , Tak V. , Nag V. L. , Varthya S. B. , and Ambwani S. , Frequency of OXA-type carbapenemases among carbapenem-resistantAcinetobacter baumanniiin clinical isolates from adult intensive care unit in India, Journal of Laboratory Physicians. (2023) 16, no. 3, 105–111, 10.1055/s-0043-1771243.

[32] Das B. J. , Singha K. M. , Wangkheimayum J. , Dhar Chanda D. , and Bhattacharjee A. , Incidence of Carbapenem-Resistant Escherichia coli ST2437 of Clinical Origin Harbouring blaOXA-144 Gene: A Report From India, Journal of Applied Microbiology. (2024) 135, no. 4, 10.1093/jambio/lxae087, 38553965. 38553965

[33] Inamdar D. P. , Inamdar P. , Annadani R. , and Basavaraju A. , Carbapenem Resistant Enterobacteriaceae: Detection by Modified Carbapenem Inactivation Method (mCIM) and Their Association With Clinical Outcome at a Teaching Hospital in Southern India, Microbes and Infectious Diseases. (2024) 10.21608/mid.2024.326507.2268.

[34] Pathak A. , Tejan N. , Dubey A. , Chauhan R. , Fatima N. , Jyoti , Singh S. , Bhayana S. , and Sahu C. , Outbreak of Colistin Resistant, Carbapenemase (blaNDM, blaOXA-232) Producing Klebsiella pneumoniae Causing blood stream Infection Among Neonates at a Tertiary Care Hospital in India, Frontiers in Cellular and Infection Microbiology. (2023) 13, 1051020, 10.3389/fcimb.2023.1051020, 36816594. 36816594 PMC9929527

[35] Caliskan-Aydogan O. and Alocilja E. C. , A Review of Carbapenem Resistance in Enterobacterales and Its Detection Techniques, Microorganisms. (2023) 11, no. 6, 10.3390/microorganisms11061491. PMC10305383 37374993

[36] Das B. J. , Singha K. M. , Wangkheimayum J. , Chanda D. D. , and Bhattacharjee A. , Emergence of Carbapenem-Resistant Enterobacterales co-Harboring blaOXA-78 and blaOXA-58 From India, Annals of Clinical Microbiology and Antimicrobials. (2023) 22, no. 1, 10.1186/s12941-023-00635-6. PMC10486080 37679795

[37] Sekar R. , Srivani S. , Kalyanaraman N. , Thenmozhi P. , Amudhan M. , Lallitha S. , and Mythreyee M. , New Delhi Metallo-*β*-Lactamase and Other Mechanisms of Carbapenemases Among Enterobacteriaceae in Rural South India, Journal of Global Antimicrobial Resistance. (2019) 18, 207–214, 10.1016/j.jgar.2019.05.028, 31181271. 31181271

[38] Abhishek S. , Naik S. S. , Leela K. V. , and Maheswary D. , Genotypic Distribution and Antimicrobial Susceptibilities of Carbapenemase-Producing Enterobacteriaceae Isolated in a Tertiary Care Hospital in South India, Journal of Pure & Applied Microbiology. (2022) 16, no. 4, 2047–2054, 10.22207/JPAM.16.4.08.

[39] Sharma S. , Banerjee T. , Kumar A. , Yadav G. , and Basu S. , Extensive Outbreak of Colistin-Resistant, Carbapenemase (blaOXA-48, blaNDM) Producing Klebsiella pneumoniae in a Large Tertiary Care Hospital, India, Antimicrobial Resistance and Infection Control. (2022) 11, no. 1, 10.1186/s13756-021-01048-w. PMC8740481 34991724

[40] Kumar A. , Mohapatra S. , Bir R. , Tyagi S. , Bakhshi S. , Mahapatra M. , Gautam H. , Sood S. , das B. K. , and Kapil A. , Intestinal Colonization due to Carbapenem-Resistant Enterobacteriaceae Among Hematological Malignancy Patients in India: Prevalence and Molecular Charecterisation, Indian Journal of Hematology and Blood Transfusion. (2022) 38, no. 1, 1–7, 10.1007/s12288-021-01415-y, 35125706. 35125706 PMC8804120

[41] Chandel N. , Gorremuchu J. P. , and Thakur V. , Antimicrobial Resistance Burden, and Mechanisms of Its Emergence in Gut Microbiomes of Indian Population, Frontiers in Microbiomes. (2024) 3, 1432646, 10.3389/frmbi.2024.1432646. 41853539 PMC12993557

[42] Modi C. M. , Singh S. P. , Pandya Y. G. , Patel C. P. , and Patel R. M. , Prevalence of Carbapenem-Resistant Enterobacteriaceae in a Tertiary Care Hospital of Gujarat, India, Journal of Clinical and Diagnostic Research. (2021) 15, no. 3, DC19–DC22, 10.7860/JCDR/2021/47332.14627.

[43] Chanda P. , Gupta S. , Chattopadhyay S. , and Bose S. , Phenotypic Detection of Carbapenem-Resistant Enterobacterales and Carbapenem-Resistant Pseudomonas aeruginosa by mCIM and eCIM and Their Ceftazidime-Avibactam With Aztreonam Synergy Profile in a Tertiary Care Hospital in Eastern India, Asian Journal of Medical Sciences. (2024) 15, no. 8, 79–84, 10.3126/ajms.v15i8.64739.

[44] Bisht T. , Rajput M. S. , and Jain K. , Prevalence of carbapenem-Resistant Hospital-Acquired Infections and Their Antimicrobial Susceptibility Pattern in a Tertiary Care Hospital, Journal of Pharmaceutical Research International. (2023) 35, no. 17, 7–15, 10.9734/jpri/2023/v35i177386.

[45] Saxena S. , Aggarwal P. , Mitra S. , Singh S. , Kaim M. , and Sharma A. , In Vitro Assessment of Newer Colistin-Sparing Antimicrobial Agents for Clinical Isolates of Carbapenem-Resistant Organisms, Journal of Infection and Chemotherapy. (2024) 30, no. 12, 1252–1258, 10.1016/j.jiac.2024.05.017. 38839032

[46] Devi L. S. , Sardar M. , and Sharma M. , Concomitant Carbapenem and Colistin Resistance Among Escherichia coli and Klebsiella pneumoniae Isolates From Patients Visiting a Hospital in Haryana India, Medical Journal of Dr. DY Patil Vidyapeeth. (2024) 17, no. 1, 160–167, 10.4103/mjdrdypu.mjdrdypu_486_22.

[47] Manohar P. , Leptihn S. , Lopes B. S. , and Nachimuthu R. , Dissemination of Carbapenem Resistance and Plasmids Encoding Carbapenemases in Gram-Negative Bacteria Isolated in India, JAC Antimicrobial Resistance. (2021) 3, no. 1, 10.1093/jacamr/dlab015, 34223092. PMC8210035 34223092

[48] Tamma P. D. , Aitken S. L. , Bonomo R. A. , Mathers A. J. , van Duin D. , and Clancy C. J. , Infectious Diseases Society of America 2023 Guidance on the Treatment of Antimicrobial Resistant Gram-Negative Infections, Clinical Infectious Diseases. (2023) ciae403, 10.1093/cid/ciad428, 37463564. 37463564

[49] Singh C. , Pandey A. , and Singh L. , Health Policy Analysis on the Containment of Antimicrobial Resistance (AMR) in India: A mixed Methods Study of Antimicrobial Stewardship as a Pivotal Intervention in Tackling AMR, Anti-Infective Agents. (2024) 22, no. 4, 8–22, 10.2174/0122113525273938231221110816.

[50] Baruah J. , Shantikumar Singh L. , Salvia T. , and Sarma J. , Antimicrobial Resistance: A Continued Global Threat to Public Health–a Perspective and Mitigation Strategies, Journal of Laboratory Physicians. (2024) 16, no. 4, 10.25259/JLP_24_2024.

[51] Farooq S. M. , Prevalence and Characterization of Multi Drug Resistant Gram Negative Bacilli Isolates From a Tertiary Care Centre of Western U.P., India, Journal of Pure and Applied Microbiology. (2019) 13, no. 2, 1069–1078, 10.22207/JPAM.13.2.45.

[52] Soanker R. , Vemu L. , Vimala S. , Kanne P. , Thumma V. M. , Chavala G. , Sudhaharan S. , and Thumma V. M. , The Effect of Double Carbapenem Regimen in the Management of Carbapenem-Resistant Klebsiella pneumoniae Infections: a Report of Five Cases, Cureus. (2024) 16, no. 1, e52023, 10.7759/cureus.52023. 38344568 PMC10854998

[53] Jain B. , Tewari S. , Richa K. , Tripathi D. N. , Dubey D. , and Paul S. , Antimicrobial Susceptibility Pattern of Newer Beta Lactam-Beta Lactamase Inhibitor Agents on the Carbapenem Resistant and Sensitive Strains of Enterobacterales and Pseudomonas aeruginosa, Indian Journal of Microbiology, Metabolism and Transmissible Diseases. (2024) 10, no. 2, 114–119, 10.18231/j.ijmmtd.2024.021.

[54] Pawar S. K. , Mohite S. T. , Shinde R. V. , Patil S. R. , and Karande G. S. , Carbapenem-Resistant Enterobacteriaceae: Prevalence and Bacteriological Profile in a Tertiary Teaching Hospital From Rural Western India, Indian Journal of Microbiology Research. (2018) 5, no. 3, 342–347, 10.18231/2394-5478.2018.0072.

