# Supplementary material for: Carbapenem Resistance in India: A Systematic Review of Epidemiology, Diagnostic Strategies, and Therapeutic Approaches
Source: Biomed Res Int. 2026 Aug 12;2026:6806493. doi: 10.1155/bmri/6806493 (PMC13463129; doi:10.1155/bmri/6806493)
Supplement: Supplementary file 1 [file BMRI-2026-6806493-s001.zip › PRISMA 2020 Checklist Carbapenem Supplementary Table 1.docx]

**PRISMA 2020 Checklist**

Supplementary Table S1 below details how this report conforms to PRISMA 2020 guidelines:

| **Section / Topic** | **Item #** | **PRISMA 2020 Checklist Item** | **Location in Report** |
| --- | --- | --- | --- |
| **Title** | 1 | Identify the report as a systematic review in the title. | Title (designated as “A Systematic Review”) |
| **Abstract** | 2 | Structured summary of objectives, methods, results, conclusions. | Abstract (structured with Background, Methods, Results, Conclusions) |
| **Introduction** | 3 | Rationale: Describe the rationale in context of existing knowledge. | Introduction (first paragraphs) – explains importance of carbapenem resistance in India. |
|  | 4 | Objectives: Provide an explicit statement of the review question(s) or objectives. | Introduction (Objectives at end) – lists the aims of this review. |
| **Methods** | 5 | Eligibility criteria: Specify inclusion and exclusion criteria for studies. | Methods – Eligibility Criteria section (defines populations, study types, date range, etc.). |
|  | 6 | Information sources: Specify all databases, registers, etc., and date of last search. | Methods – Information Sources and Search Strategy (lists PubMed, Scopus, ICMR reports; search last done April 2024). |
|  | 7 | Search strategy: Present full search strategy for at least one database, including keywords and filters. | Methods – Information Sources (provides example search terms used for PubMed). |
|  | 8 | Selection process: Describe process of selecting studies (screening, eligibility, included) and who performed it. | Methods – Study Selection (details screening and inclusion process, plus Figure 1 PRISMA flow). |
|  | 9 | Data collection process: Describe how data were extracted and any tool or form used. | Methods – Data Collection and Extraction (explains use of standardized form and double-checking). |
|  | 10 | Data items: List and define all outcomes and data items sought (e.g. prevalence, genes, etc.). | Methods – Data Collection (mentions variables extracted like resistance rates, gene presence, etc.). |
|  | 11 | Study risk of bias assessment: Specify methods for assessing risk of bias of included studies. | Methods – Quality Assessment (notes that formal scoring wasn’t done due to observational nature, but biases considered qualitatively). |
|  | 12 | Effect measures: Specify principal summary measures (e.g. risk ratio, etc.) if applicable. | Methods – (Not applicable; no meta-analysis. Stated narrative synthesis was used). |
|  | 13a | Synthesis methods: Describe methods for handling data and combining results (qualitative or quantitative). | Methods – Data Synthesis (describes narrative thematic synthesis, no statistical pooling). |
|  | 13b | Methods for handling data availability or preparation (e.g. conversions, if any). | Methods – Data Synthesis (not applicable beyond narrative; no special data transformations needed). |
|  | 13c | Methods for tabulating or visually displaying results. | Results – presented with tables/figures (two flowcharts, one checklist). Key data summarized in text and figures (PRISMA diagram, flowcharts). |
|  | 13d | Methods for synthesis – e.g. what models or software if meta-analysis. | Not applicable (no meta-analysis performed). |
|  | 13e | Methods for exploring heterogeneity (subgroup analyses, etc.). | Not applicable (no quantitative synthesis; narrative mentions differences by region/hospital instead). |
|  | 13f | Sensitivity analyses – if any done to assess robustness of results. | Not applicable (no quantitative analysis to vary). |
|  | 14 | Reporting bias assessment: Describe any methods to evaluate publication bias (e.g. funnel plot) or selective reporting. | Not formally done (most data are surveillance; we note potential under-reporting in Discussion as a limitation). |
|  | 15 | Certainty of evidence: Describe methods to assess certainty (e.g. GRADE) if done. | Not done (no GRADE; we comment on data quality qualitatively). |
| **Results** | 16a | Study selection: Give number of studies screened, assessed, included, with reasons for exclusions. | Results – Study Selection and Characteristics (reports numbers at each stage and refers to PRISMA Figure 1). |
|  | 16b | List excluded studies that seemed to meet criteria but were excluded, with reasons. | PRISMA flow (Figure 1). No specific study-by-study list due to space. |
|  | 17 | Study characteristics: For each included study, provide citation and summary of key characteristics. | Results – Study Selection and Characteristics (gives an overview of included studies, their types, scope, e.g. ICMR reports, single-center studies, etc., with references). |
|  | 18 | Risk of bias in studies: Present assessments for each study or at least discuss overall bias. | Discussed qualitatively in Methods (Quality Assessment) and touched upon in Discussion (limitations). No numeric scoring presented. |
|  | 19 | Results of individual studies: For all outcomes of interest, present for each study (e.g. effect sizes) or summary statistics. | We present key data points from individual important studies throughout Results (e.g. 28% E. coli resistance from ICMR, 84.5% OXA-48 from one study). We cite individual studies’ findings rather than a combined table due to heterogeneity. |
|  | 20a | Results of syntheses: Present summary of findings for each synthesis (e.g. for each outcome or comparison). | In Results, we structured by theme (Trends, Mechanisms, etc.), summarizing across studies. For example, “NDM and OXA-48 are predominant” is a synthesis of multiple sources. |
|  | 20b | Results of statistical analyses (meta-analyses) including heterogeneity measures. | Not applicable (no meta-analysis performed). |
|  | 20c | Results of investigations of heterogeneity (subgroup analyses). | We qualitatively describe heterogeneity (e.g. regional differences) in Geographic Patterns section. No formal statistical subgroup analysis. |
|  | 20d | Results of sensitivity analyses. | Not applicable (no quantitative analysis). |
|  | 21 | Reporting biases: Present any assessments of risk of bias due to missing results. | Not explicitly done; implicitly discussed as limitation (e.g. some data missing from East India, etc., in Discussion). |
|  | 22 | Certainty of evidence: Present assessment of certainty for each outcome (if done). | Not formally done. We note in Discussion that data are observational and may have limitations (implicitly addressing certainty). |
| **Discussion** | 23a | Interpretation: Provide a general interpretation of results in context of other evidence. | Discussion section (throughout, especially first paragraph and concluding paragraphs) – compares findings to global context, interprets rising trends, etc. |
|  | 23b | Limitations of evidence: Discuss limitations of included studies (e.g. lack of uniform data, biases). | Discussion – mentions that data are largely hospital-based, community data lacking, possible under-detection of some mechanisms, etc. (embedded in text under implications and introduction of results). |
|  | 23c | Limitations of the review process: e.g. possible missing literature, language bias. | Discussion – we note that grey literature and local reports were included to mitigate publication bias, but some unreported data may remain. We also note no formal bias scoring as a limitation in Methods. |
|  | 23d | Implications for practice, policy, and future research. | Discussion – Practical guidance is a major focus (flowcharts, etc.), and we explicitly mention need for improved access to drugs, strengthened stewardship, and surveillance (policy implications). Future research needs (e.g. new drugs, better diagnostics) are noted in concluding remarks. |
| **Other Information** | 24a | Registration: Provide registration information (if registered) or state not registered. | Methods – Protocol and Registration (states not registered). |
|  | 24b | Availability of protocol: Indicate where (if at all) protocol can be accessed. | Not applicable (no formal protocol). |
|  | 24c | Protocol amendments: Describe any amendments from the protocol. | Not applicable (no protocol). |
|  | 25 | Support: Describe sources of funding or support and role of funders. | No external funding for this review (this can be stated: “None.”). |
|  | 26 | Competing interests: Declare any conflicts of interest of authors. | None (this review was conducted impartially by the authors as an academic exercise). |
|  | 27 | Availability of data, code, and other materials: Report if any data, analytic code, etc., are available for others. | Not applicable – this review did not generate new datasets; it’s based on published sources (all of which are cited in References). |

*Supplementary Table 1: PRISMA 2020 Checklist – location of items in this report.* All essential items have been addressed to ensure transparent reporting.
