# Supplementary material for: Carbapenem Resistance in India: A Systematic Review of Epidemiology, Diagnostic Strategies, and Therapeutic Approaches
Source: Biomed Res Int. 2026 Aug 12;2026:6806493. doi: 10.1155/bmri/6806493 (PMC13463129; doi:10.1155/bmri/6806493)
Supplement: Supplementary file 1 [file BMRI-2026-6806493-s001.zip › Carbaepenem Supplementary References 17th Aug.doc]

| **Serial Number** | **Reference Number** | **Type of Study,**  **Location (Primary) of the study in India** | **Research Gap** | **Problem Statements/ Important Results** | **Summarized Abstract/Methodology** | **Insights** |
| --- | --- | --- | --- | --- | --- | --- |
| 1 | 14 | Review Article; West Bengal | - There is a lack of antimicrobial resistance (AMR) surveillance data and limited information on resistance mechanisms in Indian settings. | Rising carbapenem resistance in India threatens effective treatment of multidrug-resistant bacterial infections. | - Carbapenem resistance, particularly due to the emergence of plasmid-mediated carbapenemase genes such as KPC, NDM, IMP, VIM, and OXA-48-type, is a critical global health threat. | Carbapenem resistance in India poses a significant public health challenge, needing urgent surveillance and stewardship interventions. |
| 2 | 15 | Single centre Retrospective audit, Pune | - The study highlights a lack of comprehensive, multicentric clinical data on carbapenem resistance in India. | The resistance rates for amikacin, tigecycline, minocycline, and fluoroquinolones were 88, 82, 50, and 88% respectively. OXA-23 was the most commonly isolated enzyme (86% of the isolates produced OXA-23) followed by OXA-51 and NDM. The overall mortality was high (58%). | - The study conducted a retrospective analysis of 50 episodes of bacteremia caused by CRAB, analyses enzyme patterns and factors associated with mortality in Indian ICUs. | The routine use of the combination of polymyxin and meropenem may not offer a significant advantage over monotherapy. |
| 3 | 16 | Multicentric Retrospective audit, Various locations in India | - The study primarily focuses on the genetic profiling of resistant pathogens; clinical outcomes are less addressed. | Limited regional surveillance leads to underestimation of carbapenem resistance prevalence. Sequencing and capsular diversity analysis of the 307 *K. pneumoniae sensu stricto*isolates revealed 28 sequence types, 26 K-locus types, and 11 O-locus types, with ST231, KL51, and O1V2 being predominant. | - The study investigates the genomic profiling of carbapenem-resistant Klebsiella pneumoniae in India. The study characterized 344 retrospective *K. pneumoniae* clinical isolates collected from 8 centers across India collected in 2013–2019. | The study documents for the first time the genetic diversity of K and O antigens circulating in India. The results demonstrate the practical applicability of genomic surveillance and its utility in tracking the population dynamics of CRKP. |
| 4 | 16 | Multicentric Retrospective audit, Kolkata | - The study highlights the emergence of Enterobacter species but lacks broader national data integration. | Characterization of blood isolates of carbapenem-resistant *Enterobacter cloacae* complex (CR-ECC revealed *Enterobacter hormaechei* subsp. *xiangfangensis* as the most prevalent subsp., exhibiting resistance to almost all antibiotics currently in use and harboring diverse transmissible carbapenemase genes. | - This study investigates the molecular characteristics of carbapenem-resistant bloodstream infections in India. Vitek2 MALDI and species-specific PCR were utilized. | The study emphasizes the need for strengthened vigilance for molecular epidemiological surveillance of CR-ECC due to the presence of epidemic clones with a phylogenetically diverse and wide array of antimicrobial resistance genes in vulnerable populations. |
| 5 | 17 | Multicentric Retrospective audit, Various locations in India | - The study primarily focuses on the trends and patterns of antimicrobial resistance but lacks intervention-based solutions. | Public health policies insufficiently address carbapenem resistance challenges. The findings indicate significant (p < 0.001) monthly increases in Imipenem and Meropenem resistance for *Klebsiella*, *E. coli*, and *Acinetobacter* BSIs. Importantly, Carbapenem resistance in HA-BSIs preceded that in CA-BSIs for *Klebsiella* and *Acinetobacter* (p < 0.05). | - The study investigates the patterns and trends of antimicrobial resistance in South and Southeast Asia. | The study highlights significant increases in carbapenem resistance, emphasizing vigilant monitoring and may be crucial for achieving Sustainable Development Goals by 2030. |
| 6 | 18 | Review article, Karnataka and Andhra Pradesh | - Challenges associated with carbapenem-resistant Enterobacteriaceae are multifaceted | The review identifies key epidemiological trends, including geographic variations and risk factors associated with carbapenem-resistant Enterobacteriaceae. Mechanisms of resistance are elucidated, focusing on carbapenemase production and other related factors. | The review highlights emerging therapeutic approaches, including novel antibiotics and non-traditional approaches like phage therapy, fecal microbiota transplantation, probiotics, antimicrobial peptides, vaccines, and herbal drugs. | The research efforts to explore newer combinations and alternate approaches to treat carbapenem-resistant Enterobacteriaceae infections are crucial, the importance of stringent infection prevention and control strategies cannot be overstated. |
| 7 | 19 | Surveys and interviews, Maharashtra | Carbapenem resistance among gram-negative isolates in India up to 45%; IPC practices are suboptimal; need for improved infection control. | Evaluation of IPC practices at five hospitals reveals gaps in hand hygiene, environmental cleaning, patient isolation, and communication of CRO status; emphasizes need for targeted interventions. | High carbapenem resistance and gaps in IPC practices increase risk of CRO spread; hospitals need improved measures to prevent and contain resistant organisms. | Barriers to effective isolation and communication of CRO status exist; need for better continuity of care and improved IPC implementation strategies. |
| 8 | 20 | Cross sectional prospective study, Bhubaneshwar | 29.4% prevalence of carbapenem-resistant Enterobacterales, A. baumannii, and P. aeruginosa in Eastern India; limited treatment options; regional variation noted. | Study over seven months shows 29.4% of isolates were carbapenem-resistant, mostly Enterobacterales; recommends early screening, isolation, and contact prevention. | Increasing prevalence of carbapenem-resistant infections with limited treatment options; need for early identification and prevention measures in healthcare settings. | Larger multi-centric studies needed to understand prevalence more broadly; study does not address underlying factors of resistance or effectiveness of infection control measures. |
| 9 | 21 | Retrospective study, Mumbai | Carbapenem resistance in Mumbai at 26.5% among gram-negative bacilli; NDM predominant; longer hospital stays and higher mortality; need for infection control and antimicrobial stewardship. | Study of 2,351 isolates shows 624 carbapenem-resistant, mostly Klebsiella pneumoniae; NDM detected in 33.68%; emphasizes infection control and stewardship to curb resistance. | Carbapenem resistance threatens public health due to treatment failures and high morbidity/mortality; local epidemiology and resistance mechanisms need monitoring. | Lack of comprehensive molecular testing across regions; 16.8% of isolates had no detectable resistance genes, indicating unknown resistance mechanisms requiring further study. |
| 10 | 22 | Prospective observational study. Jodhpur | High CRE prevalence in India ICU patients; Klebsiella pneumoniae most common; CRE colonisation linked to pneumonia, longer stays, and mortality; prevention crucial. | 192 ICU patients screened; 37 colonised with CRE; 89.2% developed infections; colonisation linked to median 17-day stay and 27% mortality. | CRE infections are challenging to treat with high morbidity and mortality; colonisation often precedes infection, emphasizing prevention. | Focus limited to a single ICU; underlying factors contributing to high CRE prevalence (antibiotic use, infection control, patient demographics) not explored; comparative outcomes beyond hospital stay not analyzed. |
| 11 | 23 | Prospective study, Silchar | Carbapenem-resistant E. coli ST2437 in northeast India harboring blaOXA-144; high mortality and morbidity; urgent monitoring and containment needed. | Identification of E. coli ST2437 clinical isolate (BJD_EC180) using chromogenic agar, Rapidec Carba NP, Kirby-Bauer disc diffusion, and whole-genome sequencing; multiple resistance genes detected. | Carbapenem-resistant E. coli with blaOXA-144 poses significant health threats; urgent monitoring and containment needed to control spread. | Epidemiological factors contributing to spread not explored; clinical outcomes associated with this specific strain not comprehensively assessed. |
| 12 | 24 | Prospective study, Telangana | CP-CRE detection in southern India; 82.7% patients recovered, 3.4% died; comorbidities in 27.5%; mostly urine isolates; mCIM emphasized. | Detection of 90 CP-CRE isolates over one year; antibiogram, comorbidities, and clinical outcomes analyzed; Nitrofurantoin, Fosfomycin, gentamicin sensitivity noted. | CP-CRE poses a global threat to healthcare; effective detection needed to manage patient outcomes. | Mechanisms of resistance and genetic characterization not explored; impact of specific comorbidities on infection severity and treatment efficacy requires further study. |
| 13 | 25 | Prospective observational study, Kolkata | High CRE and CRPA prevalence (50.69%) in Eastern India; 90% MBL production; synergy observed with Ceftazidime-Avibactam (CAZ) + Aztreonam (AT); need for early detection. | 217 isolates analyzed; MBL 90%, serine carbapenemase 10%; synergy testing showed ~76% positive for CZA + AT combination; mostly from blood cultures. | CRE and CRPA pose a serious public health threat; early detection and effective treatment strategies needed. | Genetic mechanisms underlying resistance not explored; clinical outcomes of combination therapy (recovery, side effects) not assessed. |
| 14 | 26 | Prospective study, New Delhi | CROs are a major public health threat in India; phenotypic detection used; efficacy of newer antibiotics assessed. | Resistance to different carbapenems ranged from 34.1% to 79 %. Majority of the carbapenemase producers were metallo-β-lactamases (MBL) producers (75 %). | Clinical specimens were processed and bacterial identification was performed using MALDI-TOF mass spectrometry followed by antimicrobial susceptibility testing using CLSI guidelines against a plethora of newer antibiotics for CROs. | Escalating prevalence of CROs in India underscores the urgency for alternative treatment options beyond colistin. |
| 15 | 27 | Prospective study, Gurugram | Significant carbapenem resistance in E. coli (23.1%) and K. pneumoniae (52.3%) in Haryana; dual carbapenem-colistin resistance (1.7%); K. pneumoniae more resistant to non-carbapenems. | 597 isolates analyzed; carbapenemase production confirmed; dual resistance rare but concerning; highlights multidrug resistance threat. | Increasing prevalence of carbapenem and colistin resistance complicates treatment and poses a public health threat. | Genetic mechanisms of resistance not explored; clinical outcomes for dual-resistant infections not analyzed. |
| 16 | 28 | Retrospective study, Bengaluru | The information on prevalence of different genes responsible for carbapenemase production in GNB in South India is limited. | 164 samples grew *Klebsiella pneumoniae* (152), *Escherichia coli* (10), and *Enterobacter* (2). *OXA-48* and *NDM* were the most common genes responsible, with 137 (84.5%) and 95 (58.6%), respectively. Among them, 70 (43.2%) showed the presence of both genes. | In this retrospective study, samples were collected over 16 months. Carbapenem-resistant GNB underwent to Xpert Carba-R assay (Cepheid, Sunnyvale, California, USA) for the detection of five important genes responsible for carbapenemase production: *NDM, KPC, VIM, OXA,* and *IMP*. | Knowledge of the prevalence of genes responsible for carbapenemase in a particular region will help in antibiotic stewardship. Xpert Carba-R may miss unknown resistance mechanisms. |
| 17 | 29 | Prospective study, Silchar | Emergence of CRE co-harboring blaOXA-78 and blaOXA-58 in India; chromosomal localization; need for continuous monitoring. | E. coli and S. marcescens clinical isolates analyzed; genes confirmed via PCR and WGS; resistant to multiple antibiotics; horizontal transfer unsuccessful. | CRE co-harboring blaOXA-78/58 pose a significant threat; ongoing monitoring needed to prevent spread. | Mechanisms preventing horizontal gene transfer unclear; clinical implications of co-harboring multiple resistance. |
| 18 | 30 | Retrospective study, Indore | Carbapenem resistance prevalence varies (14–69%) in India; carbapenemase production major mechanism; increases mortality; need for global strategies and new antimicrobials. | 17.75% prevalence among clinical isolates in Indore; E. coli and P. aeruginosa most common; high resistance to beta-lactams and cephalosporins; emphasizes need for global containment strategies. | Rising carbapenem-resistant bacteria pose severe public health and clinical challenges; limited treatment options increase mortality. | Mechanisms of resistance and genetic factors underlying dissemination not fully understood; need further molecular research to inform diagnostics and therapy. |
| 19 | 31 | Mixed methods study, Meerut | Focuses on antimicrobial stewardship programs (AMSP) in India; highlights implementation gaps and recommendations; does not specifically address carbapenem resistance. | Mixed-methods policy report analyzing AMSP implementation, achievements, challenges; progress seen but gaps in leadership support, staffing, and diagnostic stewardship exist. | AMR in India is a major public health challenge; effective AMSP essential to combat resistance but currently under-implemented. | Lack of microbiology infrastructure and shortage of key personnel (pharmacists, infection control nurses) limit effective AMSP; impact on clinical outcomes not assessed. |
| 20 | 32 | Cross sectional study, Patna | CR-GNB prevalence 16.8% in Bihar; multiple resistance genes (blaNDM, blaCTX-M, blaSHV, blaTEM); co-occurrence common (70.6%); need targeted interventions. | 16.8% carbapenem resistance among E. coli, K. pneumoniae, A. baumannii, P. aeruginosa; WGS revealed high prevalence and co-occurrence of resistance genes. | Rising CR-GNB infections pose a significant public health threat; limited treatment options and high mortality. | Clinical outcomes and treatment efficacy not evaluated; environmental and epidemiological factors driving spread not explored; transmission dynamics need investigation. |
| 21 | 33 | Cross sectional study, Moradabad | High prevalence (58.6%) of carbapenemase-producing MDR Gram-negative bacilli in Western U.P.; blaNDM most common; leads to increased mortality and hospital stays; need for monitoring and awareness. | 510 MDR GNB isolates tested; 58.6% carbapenemase producers; blaNDM most common (24.7%); associated with higher mortality, longer hospital stay, and increased treatment costs; emphasizes monitoring and awareness. | High prevalence of carbapenemase-producing MDR GNB poses clinical challenges, increasing mortality and healthcare burden; urgent need to determine prevalence and gene distribution. | Does not explore contributing factors like antibiotic use, infection control, or socio-economic factors; lacks comparison of detection methods and assessment of clinical outcomes associated with different detection strategies. |
| 22 | 34 | Descriptive study, Nellore | CRE prevalence 21.28% in Andhra Pradesh; Escherichia coli and Klebsiella pneumoniae common; resistance linked to indiscriminate carbapenem use; need infection control and antimicrobial stewardship. | 592 Enterobacteriaceae isolates analyzed; 21.28% carbapenem-resistant; E. coli 29.26%, K. pneumoniae 16.95%; highlights need for infection control and stewardship programs. | Rising CRE prevalence causes hospital-acquired infections and limits treatment options; urgent need for identification and prevention measures in healthcare and community. | Limited to one hospital, reducing generalizability; does not investigate underlying genetic mechanisms of resistance, which could inform targeted interventions. |
| 23 | 35 | Prospective observational study, Jodhpur | CRE in Rajasthan alarming; all isolates harbor carbapenemase genes, mainly blaOXA (86%) and blaNDM (79%); treatment options limited; surveillance and infection control needed. | 14 non-duplicate clinical isolates tested (E. coli, K. pneumoniae); all had carbapenemase genes; amikacin effective in 36%; all susceptible to colistin and tigecycline; highlights emergence of blaOXA. | CRE emergence poses global public health threat with limited treatment options; need to understand resistance mechanisms in regional context. | Small sample size limits understanding of prevalence and diversity; clinical outcomes of infections not explored; larger studies needed for robust data. |
| 24 | 36 | Prospective observational study, Gurugram | Carbapenem resistance in Klebsiella pneumoniae and E. coli at 29.54%; NDM and OXA-48 genes prevalent; higher rates in ICU and male patients; need stricter surveillance and infection control. | 528 isolates analyzed; 156 (29.54%) carbapenem-resistant; K. pneumoniae 53.9%, E. coli 15.6%; NDM most common, followed by OXA-48; plasmid-mediated NDM emphasizes need for monitoring; higher mortality in resistant cases. | Increasing carbapenem resistance in K. pneumoniae and E. coli threatens patient outcomes, especially in hospitals serving international patients; urgent need for monitoring and infection control. | Alternative resistance mechanisms (efflux pumps, porins) not investigated; clonal relationships of NDM-positive isolates not established, limiting understanding of transmission dynamics. |
| 25 | 37 | Prospective study, Vellore | Few investigations into plasmid-mediated resistance in Indian isolates. | Of the 151 isolates, 71% (*n =* 107) and 68% (*n =* 103) were found to be resistant to meropenem and imipenem, respectively. The most prevalent β-lactamase gene was *bla* _NDM-1_ (*n =* 22), followed by *bla* _OXA-181_ (*n =* 21). | A total of 151 non-repetitive isolates belonging to 10 genera were collected between January 2015 and December 2016 from a diagnostic centre in Tamil Nadu. | Clinical outcomes not explored; mechanisms of horizontal gene transfer and environmental factors contributing to gene dissemination not fully analyzed. |
| 26 | 38 | Prospective study, Karad | CRE prevalence 31.77% among Enterobacteriaceae; carbapenemase production and plasmid-mediated transmission; high mortality and rapid spread in ICUs; early detection and stewardship critical. | 535 clinical isolates analyzed; K. pneumoniae and E. coli predominant; emphasizes early detection, isolation, contact precautions, restricted carbapenem use, and antimicrobial stewardship programs. | Rapid emergence and spread of CRE threatens patient outcomes and ICU infection control; need for early detection and preventive measures. | Limited to rural tertiary hospital; molecular mechanisms and genetic diversity of CRE not fully explored; broader epidemiological studies needed across regions and healthcare settings. |
| 27 | 39 | Outbreak evaluation, Varanasi | CRKp outbreak in NICU with colistin and carbapenem resistance; blaNDM and blaOXA-48; high mortality; regional differences in resistance mechanisms; need infection control and surveillance. | 45 K. pneumoniae isolates analyzed (ST5235, ST5313); environmental surveillance traced outbreak; containment included infection control measures and short hospital closure; emphasizes NICU vulnerability. | Outbreak of colistin- and carbapenem-resistant K. pneumoniae in NICU poses significant mortality risk; urgent need for infection control and antimicrobial stewardship. | Limited post-outbreak hospital monitoring; colistin use not quantified; mechanisms of colistin resistance not explored; broader impact across hospital unknown. |
| 28 | 40 | Review article, Mumbai | National strategies against AMR including carbapenem resistance; National Action Plan on AMR (2017–2021); surveillance, stewardship, and education emphasized; regional tailoring required. | Highlights Jaipur and Chennai Declarations, ICMR guidelines; focus on antibiotic policies, surveillance, infection control, laboratory enhancement, and public-private collaboration. | Growing AMR crisis in India; urgent need for national policies, surveillance programs, and infection control to combat misuse and preserve antibiotic efficacy. | Insufficient research on AMR mechanisms; limited evaluation of rapid diagnostics; lack of novel antimicrobial agent development; mostly in vitro or small-scale studies. |
| 29 | 41 | Prospective study, Mizoram | Carbapenem resistance in Mizoram: meropenem 11%, colistin 48%; blaOXA-48-like and blaCTX-M-1; need surveillance and stewardship; regional variations noted. | 141 clinical isolates analyzed from two hospitals; resistance to multiple antibiotics observed; genes blaOXA-48-like and blaCTX-M-1 identified; highlights rising resistance despite limited antibiotic access. | Insufficient data on carbapenem resistance in Northeast India; rising resistance rates pose public health challenges; need region-specific interventions. | Lack of comprehensive prevalence data; environmental and agricultural contributions to resistance not studied; further investigation needed into gene spread mechanisms. |
| 30 | 42 | Review article, Punjab | High prevalence of carbapenem-resistant Acinetobacter baumannii in ICUs; bla-OXA-23 common (92.62%); clinical challenge for pneumonia and bacteremia; molecular surveillance needed. | Study from 2013–2020 analyzed blaOXA genes; highlights oxacillinase-producing A. baumannii in immunocompromised ICU patients; need for effective management and prevention. | Carbapenem-resistant A. baumannii in ICUs poses high nosocomial infection risk; urgent surveillance and management strategies needed. | Limited understanding of hydrolysis activity of Class-D beta-lactamases; need enhanced indigenous molecular surveillance and genomic tracking. |
| 31 | 43 | Prospective study, Tamil Nadu | CRE emergence in rural South India: 3% prevalence; NDM in 49.2% of isolates; clinical impact significant; surveillance and public health measures required. | 444 non-susceptible isolates analyzed over three years; 177 tested for carbapenemase genes; NDM predominant; MIC testing emphasized for patient management. | Rising prevalence and dissemination of CRE in rural South India; need to understand mechanisms and monitor spread to protect public health. | Environmental and healthcare practice factors contributing to CRE spread not studied; genetic diversity and transmission pathways of carbapenemase genes not analyzed. |
| 32 | 44 | Observational study, multiple locations in India | AMR genes prevalent in healthy gut microbiome; geographic variations noted, especially in tribal populations; mobile genetic elements pose transmission risk. | 141 fecal samples analyzed; 188 resistance genes detected; E. coli carries most genes; highlights potential horizontal transfer and population-specific differences. | Limited data on AMR genes in healthy gut microbiomes of Indian populations; may facilitate horizontal transfer and spread of resistance. | Insufficient understanding of environmental and lifestyle factors driving geographic differences; need more comprehensive studies in healthy populations. |
| 33 | 45 | Multicentric restrospective study, Bengaluru | CRKP mortality high (68%); resistance via carbapenemases and horizontal gene transfer; genomic diversity observed; high-risk MDR clones widespread. | 344 clinical isolates from 8 centers analyzed (2013–2019); multiple sequence types and capsular types; blaOXA-48-like and blaNDM-1/5 prevalent; ST231 identified as MDR lineage. | High dissemination and mortality of CRKP in India; need for genomic surveillance and understanding of resistance and virulence mechanisms. | Limited epidemiological surveillance of MDR K. pneumoniae; incomplete understanding of capsule diversity and genetic determinants; gaps in linking genotypic diversity to clinical outcomes. |
| 34 | 46 | Prospective observational study, Chandigarh | CRKP mortality high (68%); resistance via carbapenemases and horizontal gene transfer; genomic diversity observed; high-risk MDR clones widespread. | 344 clinical isolates from 8 centers analyzed (2013–2019); multiple sequence types and capsular types; blaOXA-48-like and blaNDM-1/5 prevalent; ST231 identified as MDR lineage. | High dissemination and mortality of CRKP in India; need for genomic surveillance and understanding of resistance and virulence mechanisms. | Limited epidemiological surveillance of MDR K. pneumoniae; incomplete understanding of capsule diversity and genetic determinants; gaps in linking genotypic diversity to clinical outcomes. |
| 35 | 47 | Prospective study, Jodhpur | Th study was designed to find the frequency of oxacillinases (*OXA*) genes including *OXA-23*, *OXA-24*, *OXA-51*, and *OXA-58*. | The frequency of the *OXA-23* gene was high with 87.5%, followed by *OXA-51* gene with 73.2%. All 56 isolates were negative for the *OXA-24* and *OXA-58* genes. Both *OXA-23* and *OXA-51*genes coexisted in 40 (71.4%) isolates. | DNA was isolated from carbapenem-resistant *A. baumannii* and amplified using conventional polymerase chain reaction technique and gel electrophoresis for visualization of results. | Early detection of these drug-resistant genes by molecular methods is essential in decreasing the spread of carbapenem-resistant *A. baumannii.* |
| 36 | 48 | Outbreak evaluation, Lucknow | MDR Klebsiella pneumoniae outbreak in NICU caused high mortality; resistance via blaNDM-1, blaNDM-5, blaOXA-232; early detection, surveillance, and strict infection control crucial; regional variations necessitate tailored approaches. | Outbreak in NICU affected 5/7 neonates; isolates resistant to carbapenems and colistin; environmental screening identified K. pneumoniae ST101 in incubator water; strict infection control contained outbreak. | MDR Klebsiella pneumoniae outbreaks in NICUs pose high risk of mortality and morbidity; urgent need for early detection, surveillance, and infection control to prevent dissemination of high-risk clones. | Potential sources beyond incubator water not explored; long-term clinical outcomes for affected neonates not analyzed; limited understanding of outbreak spread within hospital. |
| 37 | 49 | Prospective study, Kerala | MDR Pseudomonas aeruginosa in South India shows high resistance (93% to meropenem); resistance via NDM (blaNDM-1) and oprD loss-of-function; monitoring and targeted therapies needed; regional variations exist. | 74.1% isolates MDR; 55.8% resistant to all tested antibiotics; novel mutations in nalD, GyrA, and mexA overexpression identified; carbapenem resistance linked to oprD mutations and NDM prevalence. | High prevalence of MDR P. aeruginosa threatens clinical outcomes; understanding molecular mechanisms is essential for designing effective treatments. | Clinical outcomes and treatment responses not studied; environmental and transmission dynamics contributing to spread not analyzed. |
| 38 | 50 | Prospective observational study, Tamil Nadu | Carbapenem resistance among Enterobacteriaceae in a South Indian tertiary care hospital is 30%; plasmid-encoded genes bla OXA-48, bla VIM, bla NDM identified; limited treatment options; early detection and antibiotic stewardship critical. | Study of 130 clinical strains revealed 30% carbapenem resistance; Klebsiella pneumoniae predominant; phenotypic and PCR methods identified OXA-48, VIM, NDM genes; emphasizes early detection and molecular surveillance to guide therapy. | Rising CRE prevalence restricts treatment options; need for early detection and tailored therapy to control multidrug-resistant infections. | Lack of high-quality epidemiology and detailed analysis of resistance gene transmission dynamics; insufficient understanding of how carbapenemase genes spread among bacterial strains and their role in multidrug resistance. |
| 39 | 51 | Cross sectional study, Nalanda | CRE colonization in hematological malignancy patients in India; high prevalence of gut colonization leads to bacteremia; genes blaNDM-1 and blaOXA-48 detected; screening and infection control needed. | Prospective surveillance of 151 patients identified 209 CRE isolates, mostly E. coli; high resistance to ertapenem; blaNDM-1 (57.3%) and blaOXA-48 (37.8%) prevalent; supports screening for gut CRE colonization to guide therapy and infection control. | Gut colonization by CRE in vulnerable patients increases the risk of bacteremia, necessitating targeted screening and preventive measures. | Focuses on prevalence and molecular characterization; lacks analysis of clinical outcomes, morbidity/mortality impact, and mechanisms of resistance transfer among bacteria. |
| 40 | 52 | Retrospective study, Lucknow | Carbapenem resistance in Enterobacterales and P. aeruginosa in India; mechanisms include carbapenemase genes (blaKPC, blaNDM); need for newer antibiotics like Ceftazidime-Avibactam (CZA); antimicrobial stewardship and localized surveillance needed. | Assessed CZA susceptibility against carbapenem-resistant and sensitive strains; CZA effective in carbapenem-sensitive isolates (90.5% Enterobacterales) but limited in resistant strains (max 18.6% CRKP); suggests judicious use and combination therapy for MBL-producing organisms. | MDROs and CROs cause “difficult to treat” infections with limited therapeutic options, emphasizing the urgent need for newer antibiotics and stewardship to mitigate resistance. | Retrospective single-center study limits generalizability; molecular detection of carbapenemase genes not included, limiting insight into resistance mechanisms and susceptibility patterns. |
| 41 | 53 | Editorial article, New Delhi | Alarming rise of AMR in India, particularly β-lactams and fluoroquinolones; carbapenem resistance contributes to mortality; six priority pathogens responsible for >70% global deaths; urgent public health interventions needed. | AMR responsible for 4.95 million deaths in 2019; projections estimate 10 million deaths worldwide by 2050 (2 million in India); resistance threatens modern medicine; emphasizes urgent measures to combat AMR. | Rising AMR leads to high mortality and threatens modern medical practices, highlighting urgent need for interventions in India. | Lack of new antibiotic development; insufficient comprehensive strategies to address AMR in India; gap in research on effective public health measures. |
| 42 | 54 | Retrospective study, Gujarat | High prevalence of CRE in tertiary care hospital, Gujarat (29.07%, 1.99/1000 patient-days); Klebsiella spp. most common; need for screening, source isolation, and strict infection control. | Five-year surveillance (2014–2018) shows CRE prevalence and incidence rates; Klebsiella most common; highlights need for infection control to reduce spread. | CRE infections are a public health concern due to limited treatment options, necessitating prevalence monitoring and control measures in hospital settings. | Study does not explore underlying factors influencing CRE rise (antibiotic use, patient demographics, hospital practices); lacks evaluation of current infection control effectiveness and specific intervention recommendations. |
| 43 | 55 | Review article, Assam | Carbapenem resistance in K. pneumoniae and A. baumannii leads to high mortality; mechanisms include enzymatic neutralization and outer membrane changes; public health strategies should include national action plans and stewardship programs. | AMR threatens global health; pathogens show multi-drug resistance and difficult-to-treat infections; need comprehensive strategies, innovative interventions, and stewardship. | AMR undermines antibiotic effectiveness, complicating treatment and increasing mortality, highlighting urgent need for novel strategies and new drugs. | Gaps in understanding full spectrum of resistance mechanisms; need for research on innovative interventions, alternative treatments, and implementation of national action plans/AMS programs. |
| 44 | 56 | Review article, New Delhi | India faces significant AMR challenges, particularly carbapenem-resistant pathogens like NDM-1; clinical impact includes high morbidity and mortality; public health strategies include National Task Force, Chennai Declaration, and National Action Plan; regional variations complicate control. | AMR threatens public health; India has high antibiotic use and drug-resistant pathogens; initiatives include National Task Force, Chennai Declaration, National Action Plan 2017; need integration of AMR education, hospital policies, and pharmaceutical R&D. | AMR is a major public health threat in India due to irrational antibiotic use and high burden of drug-resistant pathogens, necessitating comprehensive national strategies. | Current efforts lack a strong research component to guide strategies; need for pharmaceutical development of new antibiotics targeting resistant pathogens. |
| 45 | 57 | Case Series, Hyderabad | Clinical impact of carbapenem-resistant K. pneumoniae; double carbapenem regimen (ertapenem + meropenem) effective in critically ill patients; study focuses on clinical outcomes rather than public health. | Four clinical cases treated with double carbapenem regimen; 3 complete clinical cures, 1 microbiological cure; in vitro bactericidal activity confirmed. | Treatment of carbapenem-resistant K. pneumoniae infections is challenging due to limited antibiotic availability in India; double carbapenem regimen may be life-saving. | Long-term outcomes and potential resistance development not assessed; small sample size and lack of comparative studies limit generalizability. |

CA/HA BSIs: Community and hospital-acquired Blood Stream Infections; CROs: Carbapenem resistant organisms; IPC: Infection prevention and control; CP-CRE: Carbapenemase producing-Carbapenem resistant Enterobacteriaceae; CRKP: Carbapenem-resistant *K. pneumonia*.
